# What Is the Most Effective Technique for Bonding Brackets on Ceramic—A Systematic Review and Meta-Analysis

**DOI:** 10.3390/bioengineering9010014

**Published:** 2022-01-03

**Authors:** Inês Francisco, Raquel Travassos, Catarina Nunes, Madalena Ribeiro, Filipa Marques, Flávia Pereira, Carlos Miguel Marto, Eunice Carrilho, Bárbara Oliveiros, Anabela Baptista Paula, Francisco Vale

**Affiliations:** 1 Institute of Orthodontics, Faculty of Medicine, University of Coimbra, 3004-531 Coimbra, Portugal; raqueltravassos.91@gmail.com (R.T.); mcal9497@hotmail.com (C.N.); anabelabppaula@sapo.pt (M.R.); filipa.p.s.marques@gmail.com (F.M.); fppereira_@hotmail.com (F.P.); madalenaprata@hotmail.com (A.B.P.); fvale@fmed.uc.pt (F.V.); 2 Institute of Integrated Clinical Practice, Faculty of Medicine, University of Coimbra, 3004-531 Coimbra, Portugal; cmiguel.marto@uc.pt (C.M.M.); eunicecarrilho@gmail.com (E.C.); 3 Coimbra Institute for Clinical and Biomedical Research (iCBR), Area of Environment Genetics and Oncobiology (CIMAGO), Faculty of Medicine, University of Coimbra, 3004-531 Coimbra, Portugal; boliveiros@fmed.uc.pt; 4 Centre for Innovative Biomedicine and Biotechnology (CIBB), University of Coimbra, 3004-531 Coimbra, Portugal; 5 Clinical Academic Center of Coimbra (CACC), 3004-531 Coimbra, Portugal; 6 Institute of Experimental Pathology, Faculty of Medicine, University of Coimbra, 3004-531 Coimbra, Portugal; 7 Laboratory of Biostatistics and Medical Informatics (LBIM), Faculty of Medicine, University of Coimbra, 3004-531 Coimbra, Portugal

**Keywords:** adhesion, bonding, dental porcelain, glass ceramics, orthodontic bracket, shear strength

## Abstract

*Background*: There has been an increase in demand for orthodontic treatment within the adult population, who likely receive restorative treatments using ceramic structures. The current state of the art regarding the most effective method to achieve an appropriate bond strength of brackets on ceramic surfaces isn’t consensual. This systematic review aims to compare the available surface treatments to ceramics and determine the one that allows to obtain the best bond strength. *Methods*: This systematic review followed the PRISMA guidelines and the PICO methodology was used, with the question “What is the most effective technique for bonding brackets on ceramic crowns or veneers?”. The research was carried out in PubMed, Web of Science, Embase and Cochrane Library databases. In vitro and ex vivo studies were included. The methodological quality was evaluated using the guidelines for reporting of preclinical studies on dental materials by Faggion Jr. *Results*: A total of 655 articles searched in various databases were initially scrutinized. Sevety one articles were chosen for quality analysis. The risk of bias was considered medium to high in most studies. The use of hydrofluoric acid (HF), silane and laser afforded the overall best results. HF and HF plus laser achieved significantly highest bond strength scores in felsdphatic porcelain, while laser was the best treatment in lithium disilicate ceramics. *Conclusions*: The most effective technique for bonding brackets on ceramic is dependent on the type of ceramic.

## 1. Introduction

In recent years there has been an increase in demand for orthodontic treatment within the adult population. As of 2015, according to the American Association of Orthodontics, the demand within this age group has doubled over a four year period and this number is set to increase further in the future [1]. This can be attributed not only to evergrowing aesthetic concerns [2] but also to the expeditious evolution of orthodontic techniques [1]. In this age group, there is a high likelihood that an orthodontist will encounter complex restorative treatments using ceramic structures [1,2,3] due to their numerous advantages, namely biocompatibility, excellent aesthetics, reduced bacterial plaque accumulation, low thermal expansion, resistance to abrasion or fracture along with colour stability [4,5,6,7]. The most used ceramic used in dental practices are feldsphatic, lithium and zirconia [4,8].

Nonetheless, these types of restorations can reveal themselves quite complex for orthodontists, since achieving a reasonable bond strength on ceramic surfaces is challenging due to the presence of a glaze layer that hinders the adhesion process [7,8,9,10]. This is evident in the clinical practice as well with some studies having reported bracket adhesion failure rates on ceramic surfaces of around 9.8% after two years [7]. Consequently, orthodontists may encounter difficulties in achieving an optimal adhesion force on ceramic surfaces that is not only effective but also harmless [3,7], that is, an adhesion force that is resistant to orthodontic and masticatory forces while also retaining the function and aesthetics that are provided by this type of restoration after bracket debonding [3,7,10,11]. Recurrent bracket debonding reduces the success of orthodontic treatment, as it creates adverse consequences in terms of appliance efficiency, cost, treatment duration and patient’s comfort which can all be avoided by achieving adequate adhesion [4,10,12].

As a response to the referred difficulties, different conditioning methods of ceramic surfaces have emerged, whether they are mechanical, chemical or a combination of both, these are applied to change the ceramics’ properties and increase bonding strength [9,13]. Mechanical methods like sandblasting with aluminium oxide, the use of diamond burs and laser irradiation help produce micromechanical retentions. As for chemical methods, which are used to establish a porous surface on the ceramic, the most commonly used products include phosphoric acid (PhA), hydrofluoric acid (HF), silane and, as of recently, universal adhesives [1,4,8,9,10,13,14,15].

However, it is not only the ceramic surface treatment method that influences the bond strength, factors such as ceramic type, bracket material and design, light curing source, adhesive system properties and clinician’s experience are as equally important when trying to achieve the best results [4,7,8,13,15].

According to the current available literature, the most commonly used protocol for ceramic surface treatment starts with an oxide aluminium sandblasting, followed by conditioning with hydrofluoric acid, application of silane, and lastly the placement of bonding resin [10,16]. Despite being a highly successful technique in terms of adhesion strength, this protocol also presents itself with a few handicaps. This sequence is not only long and complex, but the use of hydrofluoric acid requires a very careful application due to its high corrosiveness, meaning that in the sequence of a direct contact it can lead to soft tissue necrosis [2,9,16,17]. 

The current state of the art isn’t consensual regarding the most effective and safest method to achieve a reasonable bond strength of brackets on ceramic surfaces. Several studies were performed with different ceramic types and used different surface treatment protocols. As such, it becomes necessary to gather and evaluate all the scientific information presently available to determine the best protocol.

## 2. Materials and Methods

This systematic review was drawn up in accordance with the Preferring Items for Systematic and Meta-Analyses and Meta-Analyses (PRISMA) guidelines and was registered in PROSPERO with the ID 282131 number. The Population, Intervention, Comparison and Outcome (PICO) question is outlined in Table 1. 

PICO question: What is the most effective technique for bonding brackets on ceramic crowns or veneers?

The literature search was carried out in several databases, namely PubMed (www.ncbi.nlm.nih.gov/pubmed), Web of Science Core Collection (webofknowledge.com/WOS), Cochrane Library (www.cochranelibrary.com), and EMBASE (www.embase.com). 

The last search was performed on 1 September 2021. The search formula for was the following: (bracket * OR ‘brace’/exp OR brace OR ‘orthodontic bracket’/exp OR ‘orthodontic bracket’ OR ‘orthodontic device’/exp OR ‘orthodontic device’) AND (‘dental porcelain’/exp OR ‘dental porcelain’ OR porcelain * OR ‘glass ceramics’/exp OR ‘glass ceramics’) AND (‘shear strength’/exp OR ‘shear strength’ OR ‘dental bonding’/exp OR ‘dental bonding’ OR ‘adhesion’/exp OR adhesion OR bond *). The same formula was applied was applied to the other databases. Articles published from 2011 to 2021 in English, Portuguese, and Spanish were searched.

Four independent reviewers scrutinized the studies, in accordance with defined inclusion criteria: in vitro or ex vivo studies evaluating the shear bond strength of brackets to ceramic substrate. There were included metallic, polycarbonate, sapphire, zirconia and ceramic brackets. Excluded criteria were all subtracts that differ from ceramic such as gold, amalgam, other metallic alloy, resins and polycarbonate/polycarboxylate; ex-vivo studies with enamel surfaces, polymerization techniques studies and surface characteristics studies.

Three external elements were consulted in case of doubt or in the absence of consensus. For each study the following information was extracted: author and date, study design, adhesion technique type (type, time, clinical application), porcelain type, sample size, test group and control group, bracket type, intervention test, results, and main conclusions.

Two reviewers independently assessed the methodological quality of included studies. In the case of discrepancies, a third reviewer was consulted. The methodological quality was checked using the guidelines for reporting of preclinical studies on dental materials by Faggion Jr. [18].

### Statistical Analysis

Studies were polled by surface treatment and porcelain type (either feldspathic or lithium disilicate). For each porcelain, treatments were compared using an ANOVA with post-hoc comparisons through the Mann-Whitney test with Bonferroni correction. To perform the comparisons, the sample variability was computed for each study considering the pool of studies which have analyzed the same treatment, and study weights were computed as a percentage of the total sample variance.

The IBM SPSS Statistics for Windows, Version 27.0 (IBM Corp.: Armonk, NY, USA) was used to perform the statistical analysis.

The synthetic measure based on weighted means for each treatment, as well as its variance, were used to plot the confidence intervals on a descriptive forest plot, using Excel (Microsoft Corporation, Redmond, WA, USA) and a bubble plot.

## 3. Results

**Figure 1:**
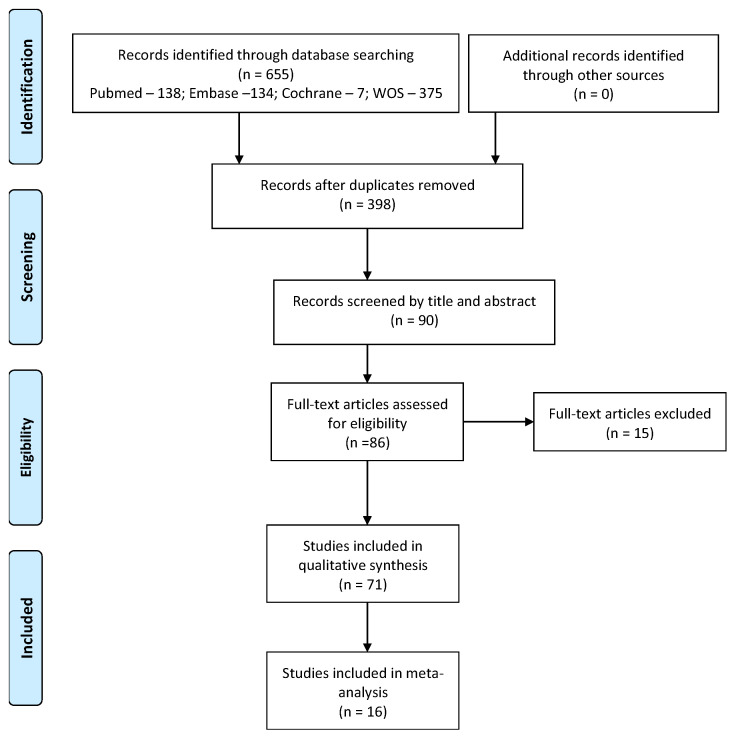
PRISMA flow diagram of studies selection.

The search results and the initial number of abstracts selected according to the selection criteria from the various databases are provided in Figure 1. From the 655 studies collected from all the databases based on their title and abstract, 90 studies were screened by title and abstract. 71 articles satisfied the final selection criteria and were included in the present systematic review and meta-analysis. Figure 1 presents the PRISMA flow of the article selection process.

The results are described in detail in Table 2. The sample size (n) ranged from 8 to 960, obtaining a total sample of n = 7246. The final selection of studies was 64 in vitro, 5 ex vivo e 2 in vitro/ex vivo, from 2011 to 2021.

All the articles evaluated various methods of conditioning the ceramic surface to obtain an adequate bond strength when bonding brackets. The types of adhesion technique mostly present in the included articles are application of orthophosphoric acid or hydrofluoric acid in various concentrations, silane application, sandblasting/air abrasion with aluminum oxide or silicon dioxide, diamond bur roughening, single bond universal adhesive and the application of different types of lasers such as Er:YAG laser, CO_2_ laser, Er:CrYSGG laser, Nd:YAG laser, Cr:YSGG laser, FS laser.

All types of porcelain (feldsphatic, lithium dissilicate glass ceramic, leucite reinforced glass ceramic, monolithic zirconia, hybrid porcelain, silica-based ceramic, lithium dissilicate-reinforced ceramic, fluoroapatite-leucite glass-ceramic, fluoroapatite, and leucite-reinforced ceramic, glazed ceramic porcelain fused to metal) were studied.

Regarding the type of brackets, metallic, ceramic, polycarbonate, sapphire, and zirconia brackets were included.

All articles used shear bond test for the application of force, except for one study that used tensile strength test [19] and another one that used the adhesion strength test [20].

### 3.1. Risk of Bias

**Figure 2:**
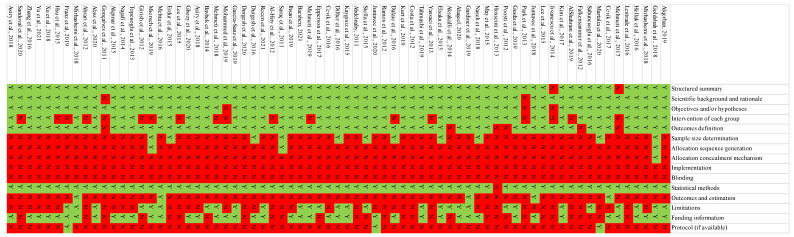
Y—yes; N—no. Risk of bias of the included studies.

The results of the quality assessment of the in vitro studies included are reported in Figure 2.

Only two studies not reported a structured abstract, calculation of the sample size [59,75] or scientific background and rationale [38,76]. Regarding the randomization process, only two studies reported these items [4,23,47]. All studies not reported researcher blinding to the interventions. Y—yes; N—no. Only a few studies reported the estimated size of outcomes [5,7,27,30,46]. No studies reported information relative to the protocol domain, except for three [15,43,74].

### 3.2. Meta-Analysis

For the quantitative analysis, only studies that used metallic brackets adhered to felspathic ceramics and lithium disilicate were selected. These studies were pooled regarding the main surface treatment used, although different protocols (concentrations, applications times, energies…) were used. Studies that presented other bracket types presented highly heterogeneous methodologies, making impossible its comparison. Also, regarding the other ceramic types, it was not possible to find studies with similar methodologies to be compared.

**Figure 3:**
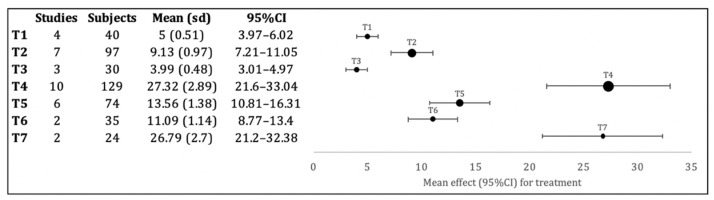
Forest plot of brackets adhesion to feldspathic ceramics with diverse superficial treatments. T1: Fine bur group; T2: Sandblasting (Al_2_O_3_) group; T3: orthophosphoric acid group; T4: hydrofluoric acid group; T5: LASER group; T6: Sandblasting (Al_2_O_3_) with hydrofluoric acid group; T7: LASER with hydrofluoric acid group. For each surface treatment, the number of studies included, the totality of samples evaluated, mean and standard deviation (SD), and 95% confidence intervals are described. Adhesion values are presented in MPa.

The meta-analysis regarding the feldspathic ceramics (Figure 3) presents the lower adhesion values for the treatments with fine bur (T1) and orthophosphoric acid (T3), without statistically significant differences between them, but significantly lower than all other treatments (*p* < 0.001). With increased adhesion values the sandblasting technique alone (T2), presents statistically significant differences (*p* < 0.001) for all groups, including the sandblasting + hydrofluoric acid group (T6), although less significant (*p* < 0.05). The group that uses LASER (T5) for surface preparation presents the following highest adhesion value with statistically significant differences (*p* < 0.001) T1, T2, T3, T4 and T7 groups and *p* < 0.05 to T5 group. The highest adhesion values were found in the LASER with hydrofluoric acid (T7) or hydrofluoric acid alone (T4) groups, without statistically significant differences between them, but being significantly higher than the others (*p* < 0.001).

**Figure 4:**
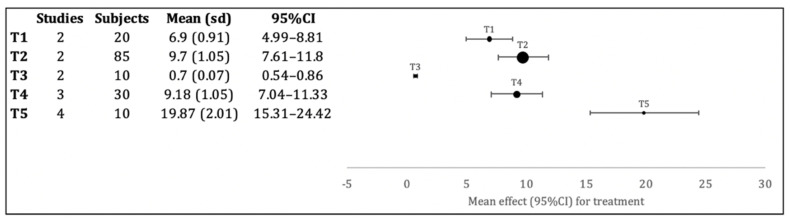
Forest plot of the evaluation of brackets adhesion to lithium disilicate ceramic with diverse superficial treatments. T1: Fine bur group; T2: Sandblasting (Al_2_O_3_) group; T3: orthophosphoric acid group; T4: hydrofluoric acid group; T5: LASER group. For each surface treatment, the number of studies included, the totality of samples evaluated, mean and standard deviation (SD), and 95% confidence intervals are described. Adhesion values are presented in MPa.

The meta-analysis that evaluates lithium disilicate ceramics (Figure 4) presents the statistically significant lowest adhesion values for the orthophosphoric acid (T3) group (*p* < 0.001). Still with low adhesion values, but higher than the previous ones, we find the fine bur group (T1), with statistically significant differences regarding all the other groups (*p* < 0.001). With increased adhesion values, we have the sandblasting technique (T2) and the hydrofluoric acid alone (T4) groups, without statistically significant differences between them, but with statistically significant differences (*p* < 0.001) with all other groups. The highest adhesion values are found in the LASER alone group (T5), with statistically significant differences from all other groups (*p* < 0.001).

For the two ceramic types evaluated in the meta-analysis, the surface presenting the lowest results is the orthophosphoric acid, with adhesion values close to 0 MPa, such as 3.99 MPa ± 0.48 for felspathic ceramics and 0.7 MPa ± 0.07 for lithium disilicate. These low adhesion results are also observed in surface treatments using only fine drill wear, with 5 MPa ± 0.51 and 6.9 MPa ± 0.91; and sandblasting with 9.13 MPa ± 0.97 and 9.7 MPa ± 1.05 for feldspathic ceramics and lithium disilicate respectively.

The treatment with the highest values for lithium disilicate ceramics is the LASER treatment with 19.87 MPa ± 2.01, while for feldspathic ceramics it is the LASER treatment with hydrofluoric acid with 26.79 MPa ± 2.7 and the treatment with hydrofluoric acid alone with 27.32 MPa ± 2.89.

When comparing the same surface treatments on the two types of ceramics, substantially different adhesion values are obtained, as an example of hydrofluoric acid with such different performances as 27.32 MPa ± 2.89 for feldspathic and 9.18 MPa± 1.05 for disilicate. The LASER treatment also presents some differences when we compare feldspathic ceramics with lithium disilicate with 13.56 MPa ± 1.38 and 19.87 MPa ± 2.01, respectively.

## 4. Discussion

The main purpose of this review was to identify the most efficient and reliable bonding protocol for orthodontic brackets to ceramic surfaces. As this is a complex and sensitive process it is essential to determine the best protocol to achieve the best results [2,4,10,12].

The last systematic review regarding this topic was published in 2014. This previous paper, that solely included in vitro studies, concluded that the best protocol would be etching with 9.6% hydrofluoric acid for 60 s, rinsing for 30 s, air-drying, and finally applying the silane [78]. With new articles emerging in recent years a new systematic review is warranted. Since we included papers published from 2011, all recent literature was scrutinized and included if relevant.

As previously stated, to ensure an acceptable shear bond strength (SBS) capable of resisting not only chewing but also forces induced by orthodontic appliances, optimal ceramic surface conditioning techniques are necessary. The present results revealed that the most studied conditioning methods include 37%/37.5% orthophosphoric acid, 4%/9%/9.5%/9.6%/10% hydrofluoric acid, silane application, sandblasting/air abrasion with aluminum oxide, diamond bur roughening, single bond universal adhesive and the use of different types of LASER, such as Er:YAG laser, CO_2_ laser, Er:CrYSGG laser, Nd:YAG laser, Cr:YSGG laser, FS laser.

### 4.1. Design and Bracket Material

The included studies present several different combinations of ceramic surface conditioning techniques to understand which one achieves a better SBS value. Some studies prove that although the ceramic surface conditioning method is the most important factor in achieving acceptable clinical values for SBS, it is not exclusive. Factors such as the material and design of the bracket, type of ceramic surface, and etch time also affect SBS. Mehmeti et al. states that the bracket type used significantly affects the SBS value and is a valid clinical concern [57]. On the other hand, Guida et al. showed that the failure rate is closely related to the glass-ceramic surface conditioning and that the bracket type is inconsequential [73]. According to Mehmeti et al., metallic brackets seemingly provide stronger adhesion with all-zirconium surfaces when compared to ceramic polycrystalline brackets, which can be attributed to their improved base surface design [59]. However, this is opposed to the findings of Al-Hity et al. which revealed that bonding strength of ceramic brackets on porcelain significantly exceeds that of metal brackets [19]. Different testing protocols and materials used can explain the contradictory results, since these two factors have a profound impact on the obtained results.

### 4.2. Orthophosphoric Acid, Fine Burr and Sandblasting

In our systematic analysis, the lowest adhesion values were verified with orthophosphoric acid, fine burr, and with slightly higher values, sandblasting treatments. Although these treatments created microroughness that could improve adhesion, their use alone presented unsatisfactory results. According to three authors (Mohammed et al., Mehta et al. and Girish et al.), the sandblasting method in association with the application of silane reaches the maximum SBS, while the use of 37% orthophosphoric acid has the lowest SBST and is deemed unsuitable for bonding ceramic brackets [21,27,31]. In this situation, we can attribute the good SBS scores to the use of silane, which alone presents high bond strength forces.

Other studies, regarding surface roughening revealed that the use of sandblasting or diamond burs along with the application of hydrofluoric acid significantly improved bond strength [52]. Sandblasting with SiO_2_ was shown to have no advantage when compared to sandblasting with AL_2_O_3_ [70].

### 4.3. Hydrofluoric Acid

The etching process partially dissolves the ceramic matrix, increasing the surface area by creating microchannels, this allows for the penetration of resin cement, thus providing finer conditions for increased bond strength.

However, since the available brands of porcelain have dissimilar particle sizes and crystalline structure, different outcomes are to be expected when testing various ceramic surfaces and brands. The heterogeneity of the reviewed studies can be attributed to structural differences in porcelain surfaces (besides the brackets’ base designs), which may result in higher or lower bond strength. As example, a paper by Kurt et al. published in 2019, reported that the highest SBS value was found in feldspathic ceramics previously treated with hydrofluoric acid [24]; however, Saraç et al. demonstrated that for any conditioning method, leucite-reinforced ceramic, in general, showed a higher SBS when compared to feldspathic and fluoroapatite ceramics [47].

As stated above, the etching agent HF increases the available surface area for adhesion. Higher HF concentrations promote more ceramic dissolution, which may be linked to higher bond strength values [79]. Such results support the use of HF as surface treatments when bonding ceramic restorations [80]. This can explain the results obtained in the feldspathic ceramics group, where the HF groups (alone or in combination with a laser) presented higher adhesion values. However, the HF promoted significantly lower adhesion values in the disilicate lithium group. Lithium silicate is more susceptible to HF action than feldspathic. HF concentrations above 5% used for more than 20 s significantly influence the characteristics of the material, promoting a decrease in the material strength [81]. Additionally, higher HF concentrations can also result in worse adhesion, as shown in an in vitro study by Pérez et al. [82].

The use of HF also produces insoluble fluorosilicate salts that remain on the material’s surface (if not removed by other methods, such as ultrasonic cleaning), which can affect the adhesion [83]. Also, the overall reduced number of studies included for this material and the different experimental methodologies used can affect the observed results. Taken together, such factors and differences in the material composition regarding feldsphatic ceramics can explain the obtained values for the disilicate lithium group.

Also, the acid etching time was inconsistent as different studies used different methodologies. According to Falkensammer et al. this factor is not preponderant for achieving SBS, according to their study an etching time of 30 s was as effective as standard conditioning (60 s) [70]. However, Costa et al. revealed that an etching time of 60 s significantly improved the SBS of brackets to feldspathic ceramic surfaces [34].

### 4.4. Silane

The use of silane improves the bond strength of brackets to ceramic surfaces [23,67]. Silane forms chemical bonds with both organic and inorganic surfaces, resulting in a stronger connection between surfaces. Furthermore, Zhang et al. reported that HF acid etching followed by silane was the best suited method for bonding on silica based ceramics and, according to Tahmasbi et al. SBS of bracket to porcelain mainly relies on the use of silane rather than the type of adhesive chosen [9,25].

### 4.5. Adhesive System

The chosen adhesive protocol will influence the bond strength of brackets to ceramic surfaces. According to the results of the studies reviewed, ceramic surfaces treated with blasting aluminum oxide followed by Single Bond Universal™ application had an improved SBS and caused less cohesive damage to the ceramic [51].

### 4.6. LASER

Recent publications studied alternatives that involve irradiating the ceramic surface with different laser types. The bond strength obtained through the combination of Er:YAG laser and HF acid on the ceramic surface may be sufficient for bonding brackets [28]. Also, according to Cevik et al. hydrofluoric acid and phosphoric acid etching methods were not suitable as surface treatment methods for feldspathic porcelains [17]. Contrarily, other studies revealed that the Er:YAG laser with the recommended settings (intensity and duration) is not a suitable alternative to the application of HF, however the laser Nd: YAG has been shown more promising results [30,65].

The results of this systematic review indicated that laser irradiation and/or HF-etching are the two surface treatments that allow greater resin-ceramic bonding. Laser irradiation emits a wavelength which is absorbed by ceramic materials, creating micro-retentions which improve resin-ceramic bonding [84]. Feitosa et al. compared 5 types of surface treatment and have found that Er:YAG laser promotes higher surface roughness, producing an improvement in the tensile strength. Regarding laser application time, these authors suggested times greater than 5 s, since some regions on the laser-treated surface had a similar morphologic appearance to the control group [85]. An article published in 2013 compared fractional CO_2_ laser with different intensities with hydrofluoric acid, showing that 10 and 15 W laser were higher shear bond strength than HF-etching with better results in deglazed specimens [29]. More recently, Mirhashemi et al. suggested that laser combined with HF promotes higher shear bond strength than laser groups only [30].

In lithium disilicate ceramic crowns, the results revealed that irradiation with different types of lasers can be effective in obtaining an adequate SBS. Conditioning with Er,Cr:YSGG and CO_2_ laser has the potential to be used in clinical settings alternative to HF+S when bonding to metallic brackets [66]. However, contrary to the previously mentioned statements, the study by Alavi et al. concluded that neither CO_2_ nor Nd:YAG lasers resulted in adequate surface changes for bonding ceramic brackets when compared to conditioned samples with HF [16]. This is also confirmed by Mirhashemi et al. who demonstrated that although conditioning with Er:CrYSGG met SBS requirements for orthodontic brackets, the SBS must be improved through refinement of the irradiation details [30]. Regarding zirconia crowns, FS laser at 200 mW and 60 μm is ideal treatment for conditioning, producing good SBS while also having a more sustainable energy consumption [53].

Importantly, no studies regarding the combined use of HF with laser (T7) included lithium disilicate ceramics, so we cannot ascertain if high bond values similar to the ones observed in the feldspathic ceramics could be obtained, or if the ceramic type is a decisive factor, like for the HF treatment.

Due to the lack of homogeneity in methodology within the currently available literature investigating the bond strength of orthodontic brackets to ceramic surfaces, the present review results present some limitations. To overcome this, calibrated studies analyzing the same parameters using the same protocols should be performed, hence providing stronger evidence. Further research focusing on surface changes, the architecture of the bracket base and the type of the adhesive resin should be performed.

## 5. Conclusions

Surface treatment protocols cannot be universal for all ceramic and/or all bracket types. Based on our results, we can conclude that for felspathic ceramics, the surface treatment which provides the best adhesion values is the use of hydrofluoric acid alone or concomitantly with LASER. For lithium disilicate ceramics, the treatment with the best results is the use of LASER alone, although combination with HF was not evaluated.

Lower bond strengths were observed in the orthophosphoric acid and fine burr groups. Further high-quality studies with similar methodologies regarding the ceramic type, surface protocol, surface changes, the architecture of the bracket base and the type of the adhesive resin are required.

## Figures and Tables

**Table 1:** The PICO question.

**Population**	Ceramic subtracts (crowns, veneers) …
**Intervention**	Adhesion Techniques …
**Comparison**	Diverse techniques (fluoride acid, sand blasting, adhesive, silane) ….
**Outcome**	Which is the most effective ….

**Table 2:** Summary of parameters and results from in vitro and ex vivo included studies.

Authors, Year	Study Design	Type of Adhesion Technique (Type, Time, Clinical Application)	Type of Porcelain	Sample Size (*n*)	Test Group	Control Group	Bracket Type	Intervention Test	Results	Conclusions
Mohammed et al., 2019 [21]	Ex vivo	Five different surface conditioning methods: G1: 37% H_3_PO_4_ acid gel (30 s) + washed + air dried + primer & bonding agent; G2: 9% HF acid (90s) G3: sandblasting for 2–3 s + 9% HF acid; G4: Sandblasting (2–3 s) + Silane, G5: Fine diamond bur roughening + silane	Porcelain	60	50 ceramic crowns fabricated onto the premolar teeth following crown preparation	Natural teeth were acid etched in conventional manner using 37% H_3_PO_4_ acid (n = 10)	Metallic	SBST	G4 produced maximum bond strength of 12.34 ± 0.95 MPa comparable or even better than the control group 11.03 ± 1.63 MPa; G2 and G3 9% HF acid 11.48 ± 0.98 MPa; G5 F 9.28 ± 1.11 MPa. Ceramic surfaces conditioned with 37% H_3_PO_4_ acid produced least SBST of 5.51 ± 0.88 MPa and hence not suitable for bonding Orthodontic brackets in a clinical scenario.	G4 produced maximum bond strength comparable or even better than the control group followed by G3 and G5. G2 produced least SBST and hence not suitable for bonding Orthodontic brackets in a clinical scenario.
Dilber et al., 2016 [22]	In vitro	Three surface conditioning methods: G1: fine diamond burr; G2: fine diamond burr + air abrasion with 30 μm SiO_2_ + silane G3: fine diamond burr +9.5% HF acid + silane	Feldspathic ceramic; Lithium disilicate glass ceramic; Nanocomposite; Polymer infiltrated ceramic network	204	CAD/CAM blocks (n = 204, n = 17 per group) of (a) VITA Mark II (VM), (b) IPS e.max CAD (IP), (c) Lava Ultimate (LU), (d)VITA ENAMIC (VE): C-Control: (fine diamond bur); CJ: (fine diamond bur + air abrasion with 30 μm SiO_2_ + silane) HF: (fine diamond bur +9.5% HF acid + silane)	Specimens were mechanically roughened with fine diamond burrs placed with their shafts parallel to the specimen axes. Then, they were washed and rinsed thoroughly to remove the debris, and air-dried	Metallic	SBST	Mean bond strength (MPa) values were significantly affected by the surface conditioning method (*p* < 0.001) but not the CAD/CAM material type (*p* = 0.052); Bond strengths for all CJ and HF-conditioned specimens were two-fold higher (11.83 ± 1.95 − 9.44 ± 1.63) than those for control specimens with all materials (4.73 ± 0.93 − 6.02 ± 0.69). Significantly lower mean values were obtained in LU-CJ (9.78 ± 1.61) and LU-HF (9.44 ± 1.63) than those for other groups (11.83 ± 1.95 − 10.93 ± 1.33) groups (*p* < 0.05).	All CAD/CAM materials tested benefitted from additional surface conditioning either with HF acid or silica coating and silanization; Weibull parameters indicated more reliable adhesion of metal brackets to feldspathic ceramic when their graze was removed with fine diamond bur and then conditioned with either hydrofluoric acid or silica coating followed by silanization compared to those of other material conditioning combinations;
Miersch et al., 2019 [23]	In vitro	(1) Roughening, etching with 9% buffered HF acid; (2) Sandblasting and silane; (3) Roughening, and an experimental single component ceramic primer containing ammonium polyfluoride and trimethoxysilylpropyl methacrylate; (4) Applying the experimental single-component ceramic primer without prior roughening; (5) Only roughening;	Leucite reinforced glass ceramic	60	60 identical molar crowns with the morphology of tooth 36 were computer-aided designed and computer-aided manufactured (CAD/CAM) from a leucite-reinforced glass ceramic. G1: roughening, hydrofluoric acid, silane; G2: roughening, silane; G3: roughening, experimental coupling agent; G4: experimental coupling agent; G5: roughening;	In group 6 (control), the buccal tube was positioned directly on the untreated ceramic surface only using the luting composite, which was polymerized by light curing (n = 10)	Metallic	SBST	The highest mean value of SBST was examined in group 1 (61.56 MPa), followed by group iii (45.53 MPa), group 2 (41.65 MPa), and group 4 (23.14 MPa). The comparison between groups 1–4 (with coupling agent) and group 5 (without coupling agent) revealed statistically significant differences (*p* ≤ 0.002), with the exception of the comparison between groups 4 and 5. Within groups 1–4, statistically significant results were determined between groups 1 and 4 as well as between groups 3 and 4 (*p* < 0.001). The SBST of group 6 was not calculated as the buccal tubes debonded after the incubation period.	A suitable coupling agent system produced clinically acceptable shear bond strengths capable of withstanding orthodontic forces.
Kurt et al., 2019 [24]	In vitro	G1: HF acid 9,6% for 2 min + silane; G2: Sandblasting with Al_2_O_3_ applied from a distance of 10 mm for 10 s in circling motions at 2.5 bar pressure + silane; G3: Silica coating with cojet under 2.5 bar pressure, at a 10-mm distance for 10 s + silane; G4: Roughening with diamond burr at 40,000 rpm for 10 s+ silane	Feldspathic porcelain monolithic zirconia hybrid porcelain	168	56 feldspathic porcelain, 56 monolithic zirconia, and 56 hybrid porcelain samples were divided into 4 surface treatment subgroups.	NR	Metallic	SBST	Of the materials conditioned with HF acid, the feldspathic porcelain group had the significantly highest bonding resistance (8.84). The surface-conditioning method did affect the SBST on different surfaces.	Variations of surface types of the materials affected the bonding resistance of orthodontic attachments. Comparisons of the materials with each other showed the highest bonding resistance to be for the feldspathic porcelain in HF acid group.
Zhang et al., 2016 [25]	In vitro	G1: 9.6% HF acid for 2 min (HF); G2: HF acid for 2 min and silane (HFS); G3: Sandblasting from a distance of 10 mm at a pressure of 3 bar for 10 s, then washed and dried for 1 min and silane (sas); G4: Silica-coating by using the intraoral sandblaster filled with 30 mL silica-modified aluminum trioxide at 3 bar pressure, from a distance of 10 mm for 10 s and silane was applied afterward (sis).	Silica based ceramic	80	G1 (HF); G2 (HFS); G3 (sas); G4 (sis).	NR	Metallic	SBST	The HF-acid-treated group revealed the lowest bond strength value (3.1 MPa), which was significantly lower than those of the other three groups (*p* = 5.82 9 10–13). Silica-coating with silane (12.3 MPa) and sandblasting with silane (11.6 MPa) groups yielded similar bond strengths (*p* = 0.14), and both showed significantly higher shear bond strength than that of the HF acid with silane group.	Shear bond strengths exceeded the optimal range of ideal bond strength for clinical practice, except for the isolated HF group. HF acid etching followed by silane was the best suited method for bonding on IPS Classic.
Recen et al., 2021 [26]	In vitro	Four surface conditioning methods: G1: cojet sand from a 10 mm distance at a pressure of 0.25 MPa for 15 s.; G2: MEP was applied and agitated into the FC surface for 20 s; G3: 9% HF acid etching for 90 s. Followed by silane coupling agent for 60 s; G4: Diamond burr for 3 s followed by silane coupling agent for 60 s.	Feldspathic porcelain	40	G1: Sandblasting; G2: Monobond^®^ Etch & Prime (MEP); G3: 9% HF and Silane coupling agent; G4: Roughening and silane.	NR	Metallic	SBST	No statistically significant difference (*p* > 0.05) was found in SBST between the groups	Considering the mean SBST values, all treatment methods except use of a diamond bur followed by a silane coupling agent can all be used for the bonding of metal brackets to the FC restorations with sufficient SBST for clinical performance. The clinical application of MEP has been found promising since it presented with comparably high SBST values to cojet and HF with safe ARI scores. Also, it eliminates the need for extra steps, minimizing the probability of contamination or the necessity to purchase additional instruments but also excludes potential detrimental effects of HF or sandblasting.
Mehta et al., 2016 [27]	In vitro	Hydrofluoric acid 4% (HF), porcelain conditioner silane primer, reliance assure primer, reliance assures plus primer, and z prime plus zirconia primer	Feldspathic porcelain and zirconia	72	36 zirconia specimens divided into 2 groups: G 1- sandblasting + HF + silane + ra primer; G 2- sandblasting + silane + ra plus primer. 36 glazed feldspathic porcelain specimens divided into two groups: G1- sandblasting + z prime plus primer; G2- sandblasting + ra plus primer.	One control group for zirconia porcelain group (sandblasting + porcelain conditioner (silane)) and one control group for feldspathic porcelain group (sandblasting + porcelain conditioner (silane))	Metallic	TBST	No statistically significant mean differences were found in tbs among the different bonding protocols for feldspathic and zirconia, 𝑝 values = 0.369 and 0.944, respectively.	Silanization following sandblasting resulted in tensile bond strengths comparable to other bonding protocols for feldspathic and zirconia surface.
Xu et al., 2018 [28]	In vitro	G1 9% HF acid for 2 min; G2 and G3 Er:YAG laser with two energy parameters: 250 mJ, 20 Hz and 300 mJ, 20 Hz; G4 and G5 Er:YAG laser with two energy parameters: 250 mJ, 20 Hz and 300 mJ, 20 Hz + 9% HF acid for 2 min	NR	90	90 ceramic chips were divided into five groups (n = 18 each):	NR	NR	SBST	The SBST in G2 and G3 (treated by laser only) were low, only 2.97 and 3.11 MPa respectively; it was 5.28 MPa in G1 (HF). The SBST of G4 and G5, treated by both laser and HF, were 6.73 and 7.09 MPa respectively, much more than G1, G2, and G3. Based on the comparison between G1 and G2, there is a statistical difference in SBST (*p* < 0.05). By comparing G1 and G3, the SBST has statistical difference (*p* < 0.05). The comparison between G2 and G4 indicates the statistical difference in SBST (*p* < 0.05). Moreover, the statistical difference in SBST exists between G2 and G5 (*p* < 0.05), G3 and G5 ( *p* < 0.05).	The exclusive use of HF acid, or Er:YAG laser could not achieve sufficient bracketing bonding strength. The bonding strength of combination strategy of 250 mJ, 20 Hz Er:YAG laser and HF acid on porcelain restoration surface can be satisfied for orthodontic bracket bonding.
Ahrari et al., 2013 [29]	In vitro	G1, G2, G3: CO_2_ laser for 10 s a silane coupling agent was applied before bracket bonding; G4: 9.6% hydrofluoric HF acid gel was used for 2 min.	Feldspathic porcelain	80	Four groups of 20: the specimens in G 1 to G3 were treated with a fractional CO_2_ laser for 10 s using 10 mJ of energy, frequency of 200 Hhz and powers of 10 W (G1), 15 W (G2) and 20 W (G3). In G4: a 9.6% hydrofluoric HF acid gel was used for 2 min.	NR	NR	SBST	Deglazing caused significant increase in SBST of laser treated porcelain surfaces (*p* < 0.05 but had no significant effect on SBST when HF acid was used for etching (*p* < 0.137). ANOVA revealed no significant difference in SBST values of the study groups when glazed surfaces were compared (*p* < 0.269). However, a significant between group difference was found among the deglazed specimens (*p* < 0.001). Tukey test revealed that the bond strengths of 10 W and 15 W laser groups were significantly higher than that of the HF acid group (*p* < 0.05).	Application of 9.6% hydrofluoric acid produced bond strength values that surpassed the minimum strength required in clinical conditions, either used on glazed or deglazed porcelain; due to the significantly higher bond strength, porcelain treatment with a fractional CO_2_ laser could be recommended as a suitable alternative technique to HF acid for bonding orthodontic brackets to deglazed feldspathic porcelain.
Mirhashemi et al., 2018 [30]	In vitro	G1: 9% HF for 2 min; G2: etching with the 9% HF for 2 min followed by irradiation with the Er:CrYSGG laser for 10 s; G3: etching with the 9% HF for 2 min followed by irradiation with the Er:YAG laser for 10 s; G4: irradiation with the Er:CrYSGG laser for 10 s without acid etching; G5: irradiation with the Er:YAG laser	Feldspathic porcelain	60	60 specimens of maxillary incisor crown were prepared and randomly assigned to five groups: G 1: etching with the 9% HF. G2: etching with the 9% HF + Er:CrYSGG laser; G3: etching with the 9% HF + Er:YAG laser; G4: Er:CrYSGG laser G5: Er:YAG laser	NR	Metallic	SBST	The average SBST [mean ± SD)] values in the five groups were as follows: HF (32.58 ± 9.21 MPa), Er:CrYSGG + HF (27.81 ± 7.66 MPa), Er:YAG + HF (23.08 ± 9.55 MPa), Er:CrYSGG (14.11 ± 9.35 MPa), and Er:YAG (6.30 ± 3.09 MPa). A statistically significant difference in SBST existed between the first three groups and the two laser groups (df = 4, F = 18.555, *p* < 0.001).	The Er:YAG laser with the stated specifications is not a suitable alternative to HF etching. In the case of Er:CrYSGG laser, although the conditioning outcome met the bond strength requirement for orthodontic brackets (that is, 6–8 MPa). Therefore, the bond strength must be further improved by fine-tuning the irradiation details.
Alavi et al., 2021 [16]	In vitro	G1: 9.6% hydrofluoric acid HF; G2: neodymium-doped yttrium aluminium garnet (Nd:YAG) laser; G3: carbon dioxide (CO_2_) laser; The glass ceramic surfaces were primed with a silane, and the brackets were bonded using a light-cured composite resin.	lithium disilicate–reinforced ceramic	36	36 lithium disilicate ceramic blocks were assigned to three groups (n = 12): G1: 9.6% HF; G2: neodymium-doped yttrium aluminium garnet (Nd:YAG) laser; G3: carbon dioxide (CO_2_) laser	NR	Metallic	SBST	The median and interquartile range of SBST values in three groups were 6.48 (1.56–15.18), 1.26 (0.83–1.67), and 0.99 MPa (0.70–2.10), respectively.	Neither CO_2_ nor Nd:YAG lasers resulted in adequate surface changes for bonding of brackets on ceramics compared with the samples conditioned with HF.
Girish et al., 2012 [31]		G2: Bur for 10 s; G3: hydrofluoric acid HF; G4: sandblasting for 10 s; G5: bur for 10 s + silane; G6: Hydrofluoric acid + silane; G7: sandblasting+ silane	NR	70	G2: bur; G3: hydrofluoric acid HF; G4: sandblasting; G5: burr+silane; G6: hydrofluoric acid HF + silane; G7: sandblasting+ silane.	G1- untreated surface (n = 10)	Metallic	SBST	Sandblasting with silane produced the highest SBST among all the groups and showed a mean value of 15.18 MPa. The weakest SBST was seen in the control group with a mean of 1.57 MPa. The statistical results showed that there was a significant difference between all the groups.	Sandblasting with silane combination produced the highest SBST, so it is a clinically suitable method for bonding orthodontic metal brackets onto ceramic surface.
Ji-Yeon Lee et al., 2015 [32]	In vitro	G0: No-primer (np); G1:porcelain conditioner (pc); G2: z-prime plus (zp); G3: monobond plus (mp); G4: zirconia liner premium (zl)	Zirconia	100	Four primer groups (n = 20 per group), and each primer was divided into two subgroups (n = 10 each) to examine by thermocycling protocols.	1 control group (np) (n = 20)	Metallic	SBST	The SBST of all experimental groups decreased after thermocycling. Before thermocycling, the SBST was G4, G2 ≥ G3 ≥ G1 > G0 but after thermocycling, the SBST was G4 ≥ G3 ≥ G2 > G1 = G0 (*p* > 0.05).	Surface treatment with a zirconia primer increases the SBST relative to no-primer or silane primer application between orthodontic brackets and zirconia prostheses.
Ihsan et al., 2019 [33]	In vitro	G1: transbondtm XT primer; G2: single bond universal adhesive for 20 s, and also air dried for 5 s, and then light cured for 10 s; G3: theracem, was done in the same way as described with the previous groups except that no priming or bonding agent to the zirconia surfaces was needed according to manufacturer instructions.	Zirconia	30	Single bond universal adhesive group (n = 10); Theracem group (n = 10).	G1: control group (n = 10)	Metallic	SBST	The highest value of the mean shear bond strength was in G2 (16.299 ± 2.201 MPa), followed by that of G3 (15.373 ± 1.575 MPa), while the G1 had the lowest value (5.337 ± 1.274 MPa). ANOVA showed that there was a statistically highly significant difference (*p* ≤ 0.01) among the mean values of the shear bond strength of all groups.	The two types of 10-mdp-containing adhesive systems provide good value of shear bond strength for buccal tubes bonded to zirconia surface, however, single bond universal adhesive/composite resin is the best.
Mehmeti et al., 2019 [8]	In vitro	Two different etching materials were used for conditioning of the surface of ceramic crowns: 5% HF and 37% H_3_PO_4_ for 120 s, and subsequently silane.	Zirconia and lithium-disilicate ceramics	96 (all-ceramic crowns, of which 48 full contour zirconia and 48 lithium disilicate)	Eight groups: G1: Metallic bracket bonded to zirconia surface etched with H_3_PO_4_; G2: Metallic bracket bonded to zirconia surface etched with HF; G3: Ceramic bracket bonded to zirconia surface etched with H_3_PO_4_; G4: Ceramic bracket bonded to zirconia surface etched with HF; G5: Metallic bracket bonded to lithium disilicate surface etched with H_3_PO_4_; G6: Metallic bracket bonded to lithium disilicate surface etched with HF; G7: Ceramic bracket bonded to lithium disilicate surface etched with H_3_PO_4_; G8: Ceramic bracket bonded to lithium disilicate surface etched with HF.	NR	Ceramic and metallic orthodontic brackets	SBST	Lithium-disilicate showed better bond strength in almost all groups. However, no significant difference between the groups was noticed and none of the factors had a significant influence on the mean values of SBST (*p* > 0.05).	The use of HF for surface etching of zirconia and lithium-disilicate, does not cause a significant increase in the SBST values as compared to etching with H_3_PO_4_ and silane application.
Costa et al., 2012 [34]	In vitro	G1 and G2: 10% hydrofluoric acid gel for 20s with or without silane; G3 and G4: 10% hydrofluoric acid gel for 60s with or without silane.	Feldspathic porcelain	8	G1 and G2: cylinders were etched using 10% hydrofluoric acid gel for 20 s only (n = 2) and 10% hydrofluoric acid gel for 20 s and silane (n = 2); G3 and G4: cylinders were etched using 10% hydrofluoric acid gel for 60s only (n = 2) and 10% hydrofluoric acid gel for 60s and silane (n = 2).	NR	Metallic	SBST	Silane application increased bond strength significantly (*p* < 0.05) compared with no silane application; the bonding material transbond XT promoted a significantly higher (*p* < 0.05) shear bond strength than fuji ortho lc, with or without silane application and for both etching times. The specimens etched for 20 s showed significantly lower (*p* < 0.05) shear bond strengths than those etched for 60s, for both bonding materials.	Etching time of 60 s, application of silane and transbond XT resin significantly improved the shear bond strength of brackets to feldspathic ceramics.
Dalaie et al., 2016 [35]	In vitro	9% hydrofluoric acid for 2 min and silane	Feldspathic porcelain	40	40 porcelain-fused-to-metal restorations and four different bracket base designs were bonded to these specimens	NR	Metallic and ceramic	SBST	One-way ANOVA showed that the SBST values were significantly different among the four groups (*p* < 0.001). Groups 1, 2, and 4 were not significantly different, but group 3 had significantly lower SBST (*p* < 0.001).	The bracket base design significantly affects the SBST of brackets to feldspathic porcelain.
Juntavee et al., 2020 [15]	In vitro	9.6% HF for 15 s	Feldspathic based ceramic; lithium disilicate glass-ceramic; fluoroapatite-leucite glass-ceramic; BIS-GMA, BIS-EMA, TEGDMA 73–77% silanated quartz and silica; UDMA, TEGDMA, sodium fluoride, 85% fused silica; uncured methacrylate monomer, inert material fillers, fused silica.	60	Machined ceramic specimens (10 × 10 × 2 mm) were prepared from vitablocs mark II (vita) and IPS e.max^®^ CAD (ivoclar). Layered porcelain fused to metal was used to fabricate PFM specimens. Half of specimens (n = 30) were etched. Three resin bonding systems were used for attaching metal brackets to each group (n = 10): transbond™ XT (3 m), light bond™ (reliance), or blugloo™ (Ormco), all cured with LED curing unit.	Control group (n = 30) specimens nonetched	Metallic	SBST	There were significant effects on SBST of metal bracket to the ceramic veneering materials due to the factor of different types of ceramic materials, surface treatment, resin bonding materials, interaction between types of ceramic materials, and types of adhesive resin cement (*p* < 0.05). The mean SBST of metal bracket bonded to vitablocs™ mark II was higher than bonded to IPS e.max^®^ CAD and bonded to IPS d.SIGN^®^ porcelain (*p* < 0.05). The mean SBST of metal bracket bonded to IPS d.SIGN^®^ porcelain for PFM was significant lower than the mean SBST of metal bracket bonded to vitablocs™ mark II ceramic materials (*p* < 0.05). Also, the mean SBST of metal bracket bonded to IPS e.max^®^ CAD ceramic reveals significantly lower than the mean SBST of metal bracket bonded to vitablocs™ mark II ceramic materials (*p* < 0.05).	Etching ceramic surface enhanced ceramic-bracket bond strength. However, bond strengths in nontreated ceramic surface groups were still higher than bond strength required for bonding in orthodontic treatment.
Kaygisiz et al., 2015 [36]	In vitro	G1: Sandblasting with AL_2_O_3_ for 4 s; G2: Er:CrYSGG laser; G3: sandblasting + etching with HF + silane; G4: etching with HF + silane	Three groups: metal, sapphire and zirconia (n = 28/group).	84	The mounted specimens were randomly divided into four groups: G1: sandblasting with Al_2_O_3_; G2: Er:CrYSGG laser; G3: sandblasting, etching with HF and silane, G4: etching with HF and silane application) (n = 7/group)	NR	Metallic, saphire and zirconia	SBST	Statistical analysis indicated significant differences among surface treatment procedures (*p* < 0.0001). In addition, the effect of the first and second bonding factors on SBST behaviors was shown to be significant for the brackets (*p* < 0.001).	The use of sandblasting, HF treatment and silanization procedure could be used for improving the rebond shear bond strength of zirconia brackets to porcelain surface.
Topcuoglu et al., 2013 [37]	Ex vivo	Sandblasted + 9.6% HF gel for 2 min; Er:YAG laser short pulse (sp); Er:YAG laser super short pulse (ssp); sandblasted+ sp, or sandblasted + ssp	Porcelain-fused-to-metal	150	Nine groups differing in adhesive system and surface treatment. In five groups, the adhesive system was Relyx u 200 and in the other four, Transbond XT was used. For each adhesive system, the porcelain surfaces were treated in one of five different ways: sandblasted + HF, Er:YAG laser sp, Er:YAG ssp, sandblasted + sp, or sandblasted + ssp.	Sandblasted group with transbond XT (n = 15)	Metallic	SBST	There were statistically significant differences among groups (*p* = 0.002). The highest SBST were observed in G2 (8.83–3.3 MPa), followed by groups 1, 8, 10, and 9 (in that order) with values of 8.25–3.2, 3.48–1.7, 3.11–0.93, and 1.56–0.86 MPa, respectively. The results of the independent samples t-test indicated that there were no statistically significant differences between G1 and the control group (*p* = 0.635). There were no statistically significant differences between G8 and G10 (*p* = 0.502).	Er:YAG laser application did not allow for elimination of the hydrofluoric acid step.
Gonçalves et al., 2011 [38]	In vitro	G1: 10% hydrofluoric acid for 20 s + silane; G2: 10% hydrofluoric acid for 60 s + silane (after application of silane on the ceramic surface, metallic brackets were bonded to the cylinders using Transbond XT).	Feldspathic ceramic	60	The specimens for each etching time were assigned to four groups (n = 15), according to the light source: xl2500 halogen light, Ultralume 5 LED, Acucure 3000 argon laser, and Apollo 95e plasma arc. Light-activation was carried out with total exposure times of 40, 40, 20 and 12 s, respectively.	NR	Metallic	SBST	Specimens etched for 20 s presented significantly lower bond strength (*p* < 0.05) compared with those etched for 60 s.	Only the etching time had significant influence on the bond strength of brackets to ceramic.
Akpinar et al., 2015 [39]	In vitro	G1: SB for 3 s with Al_2_O_3_; G2: 9.6% HF gel for 4 min (hf); G3: Nd:YAG laser irradiation (ny) for 20 s; G4: performance of femtosecond laser pulses; after surface conditioning, all specimens were cleaned for 380 sec in an ultrasonic cleaner and were air dried in air stream before bonding.	Feldspathic porcelain	80	G1: Group sandblasting; G2: group HF; G3: group any LASER; G4: performance of femtosecond laser pulses (each group, n = 20)	NR	Metallic	SBST	The bond strength in G3 (5.11–1.53) was significantly lower than the other groups (*p* < 0.05). There were no statistically significant differences among G1 (9.07–3.76), G2 (9.09–3.51), and G4 (11.58–4.16) (*p* = 0.28).	G4 treatment produced high SBST of the processes assessed; therefore, it appears to be an effective method for bonding orthodontic metal brackets to prepared porcelain surfaces.
Asiry, et al., 2018 [40]	In vitro	G1: HF; G2:deglazing using diamond burr (DB); G3: sand blasting (SB) with 25 μm aluminum trioxide (Al_2_O_3_); G4: tribochemical silica coating (TS) with 30 μm silica coated aluminum trioxide (Al_2_O_3_)	NR	120	Four groups of 30 specimens: G1: group HF; G2: group DB; G3: group SB; G4 group TS; 15 specimens from each group were subjected to thermocycling and the remaining 15 specimens served as the baseline (n = 15).	60	NR	SBST	Group 4 exhibited highest SBST at baseline (14.68 ± 0.28) and after thermo-cycling (12.67 ± 0.22) while G1 specimens exhibited lowest SBST at baseline (6.32 ± 0.15) and after thermo-cycling (4.32 ± 0.26). G1 specimens demonstrated lowest SBST; and G4 specimens showed the highest SBST.	Increased surface roughness enhanced SBST of the specimens.
Erdur et al., 2015 [41]	In vitro	G1: Sandblasting for 20 s; G2: etching with 5% HF acid for 20 s;G3: Nd:YAG laser for 20 s; G4: Er:YAG laser for 20 s; G5: Ti:sapphire laser; After conditioning all ceramic surfaces, silane was applied to the ceramic surfaces.	Feldspathic and IPS Empress e-Max	150	150 ceramic discs were prepared and divided into two groups. In each group, the following five subgroups (n = 15) were set up: G5 Ti:sapphire laser, G3: Nd:YAG laser, G4: Er:YAG laser, G1: sandblasting, and G2: HF acid.	NR	NR	SBST	Feldspathic and IPS Empress e-Max ceramics had similar SBST values. The Ti:sapphire femtosecond laser (16.76–1.37 MPa) produced the highest mean bond strength, followed by sandblasting (12.79–1.42 MPa) and HF acid (11.28–1.26 MPa). The Er:YAG (5.43–1.21 MPa) and Nd:YAG laser (5.36–1.04 MPa) groups were similar and had the lowest SBST values.	Ti:sapphire laser- treated surfaces had the highest SBST values. Therefore, this technique may be useful for the pretreatment of ceramic surfaces as an alternative to conventional’ techniques.
Asiry et al., 2018 [42]	In vitro	G1: IPS ceramic etching gel™ and Monobond plus™; G2: Monobond etch and prime™.	Lithium disilicate	40	The specimens were randomly assigned to two experimental groups (n = 20), G1 specimens were treated with two-step surface conditioning system (IPS ceramic etching gel™ and Monobond plus™) and G2 specimens were treated with one-step surface conditioning system (Monobond etch and prime™). Ten randomly selected specimens from each group were subjected to thermo-cycling and the remaining ten served as baseline.	N = 20	NR	SBST	The specimens treated with two-step conditioning system had higher bond strength than one-step conditioning system.	Traditional two-step conditioning provides better bond strength. The clinical importance of the study is that, the silane promoted adhesion significantly reduces on exposure to thermo-cycling.
Franz et al., 2019 [43]	In vitro	The bonding agent G1: Monobond S (Ivoclar Vivadent) or G2: Monobond Etch & Prime	Zirconia	20	The ceramic blocks (n = 20) were randomized and divided into two groups and fixation of brackets was done either by using the bonding agent Monobond S (Ivoclar Vivadent) or Monobond Etch & Prime	NR	Metallic	SBST	SBST resulted in significantly higher shear bond values when Monobond Etch & Prime was used compared to the use of Monobond S.	The use of Monobond Etch & Prime has great potential for the bonding of brackets on dental zirconia ceramics.
Yu et al., 2021 [44]	In vitro	All specimens were etched with 9.5% hydrofluoric acid for 20 s. The etched specimens were randomly assigned to one of four groups according to the adhesive used and the use (or not) of additional silane pretreatment for 20 s.	Lithium disilicate glass ceramic	80	Four groups (n = 20) defined by the pretreatment and adhesive used: G1 Adper Single Bond 2 (SB2); G2 silane + Adper Single Bond 2 (S@SB2); G3 Single Bond Universal (SBU); and G4 silane + Single Bond Universal (S@SBU).	NR	Metallic	SBST	In all groups, the mean SBST values were statistically significantly lower (*p* < 0.001) after thermocycling than before. Furthermore, specimens in groups S@SB2 and S@SBU, both of which had silane pretreatment, showed statistically significantly higher mean SBST values than did the corresponding groups without silane pretreatment (*p* < 0.05).	The application of a silane-containing universal adhesive without silane pretreatment achieves adequate durability of the bond of metal brackets to dental glass ceramics
Abdelnaby, 2011 [45]	In vitro	G1: 9.6% HF for 2 min; G2: 9.6% HF for 2 min + silane; G3: sandblasting for 10 s+ 9.6% HF for 2 min; G4: sandblasting for 10 s+ 9.6% HF for 2 min+ silane.	Feldspathic porcelain	100	The specimens were divided into four equal groups (n = 25). Porcelain surfaces were conditioned with different protocols. In G1, hydrofluoric acid and Embrace First-Coat primer were used. G2, hydrofluoric acid and silane were utilized. G3 and G4, sandblasting with aluminum oxide powder was done instead of etching.	NR	Metallic	SBST	Embrace First-Coat and silane exhibited a comparable SBST. The sandblasting process significantly increased SBST. No significant difference was found in bond SBST utilizing either hydrofluoric acid and Embrace First-Coat or sandblasting and silane. With regard to CSBS, the use of sandblasting and Embrace First-Coat revealed the highest significant CSBS value, followed by sandblasting and silane. Etching with hydrofluoric acid prior to application of either primer exhibited the least CSBS values; however, no significant difference was found between them. The SBST was significantly higher than CSBS.	Embrace First-Coat primer could be used successfully as an alternative to silane. Sandblasting provides higher bond strength than did hydrofluoric acid. Cyclic loading significantly decreased bond strength.
Cevik et al., 2017 [46]	In vitro	G1: 37.5% orthophosphoric acid for 4 min; G2: 9.6% hydrofluoric acid for 3 min; G3: Nd:YAG laser irradiation for 30 s.; G4: sandblasting with 50 μm Al_2_O_3_ particles for 10 s; G5: grinding with a diamond bur for 30 s. All samples were primed with silane before the bracket bonding, including the control group.	Feldspathic and lithium disilicate	120	Five subgroups depending on surface treatment (n = 10) G1: 37.5% orthophosphoric acid; G2: 9.6% hydrofluoric acid; G3: Nd-YAG laser irradiation; G4: sandblasting with 50 μm Al_2_O_3_ particles; G5: grinding with a diamond bur.	Control group (n = 10)	Metallic	SBST	G4 demonstrated significantly higher shear bond strengths than other groups	Surface conditioning methods, except for sandblasting and grinding, were associated with lower shear bond strengths; however, thermocycling may have had negative effects on bond strengths of specimens. Furthermore, in each ceramic system, there was a significant difference between surface-conditioning methods and surface roughness with regard to shear bond strength.
Saraç et al., 2011 [47]	In vitro	G1: Air-particle abrasion (APA) with 25 mm for 4 s Al_2_O_3_; G2: silica coating with 30 mm Al_2_O_3_ particles modified by silica for 4 s. Then silane application.	Feldspathic, fluoro-apatite, and leucite-reinforced ceramic.	60	20 feldspathic, 20 fluoro-apatite, and 20 leucite-reinforced ceramic specimens were examined following two surface-conditioning methods: G1: APA with 25 mm Al_2_O_3_ and G2: silica coating with 30 mm Al_2_O_3_ particles modified by silica.	NR	Metallic	SBST	The lowest SBST was with APA for the fluoro-apatite ceramic (11.82 MPa), which was not significantly different from APA for the feldspathic ceramic (13.58 MPa). The SBST for the fluoro-apatite ceramic was significantly lower than that of leucite-reinforced ceramic with APA (14.82 MPa). The highest SBST value was obtained with silica coating of the leucite-reinforced ceramic (24.17 MPa), but this was not significantly different from the SBST for feldspathic and fluoroapatite ceramic (23.51 and 22.18 MPa, respectively). The SBST values with silica coating showed significant differences from those of APA.	Chairside tribochemical silica coating significantly increased mean bond strength values; With all surface-conditioning methods, leucite-reinforced ceramic, in general, showed a higher SBST than feldspathic and fluoro-apatite ceramics.
Hsu et al., 2015 [48]	In vitro	G1: 37% phosphoric acid solution for 60 s, and porcelain primer (H_3_PO_4_) was applied to the etched porcelain crown surface for another 60 s; G2: 9% HF acid solution for 60 s and silane for 60 s.; G3: 9% HF solution for 60 s and generic/pentron silane for 60 s; G4: 37% phosphoric acid etching solution for 60 s and ultradent silane for 60 s; G5: 37% phosphoric acid etching solution for 60 s and generic/pentron silane for 60 s.	Glazed ceramic porcelain fused to metal (PFM)	50	Five groups for bonding, each group n = 10; G1 (H_3_PO_4_-Porcelain Primer group); G2 (HF-Ultradent Silane); G 3 (HF-Jeneric/Pentron Silane); G 4 (H_3_PO_4_-Ultradent Silane)Ultradent; G 5 (H_3_PO_4_-Jeneric/Pentron Silane).	NR	Metallic	SBST	The Porcelain Primer group had the lowest bond strengths and the H_3_PO_4_-Jeneric/Pentron silane group had the highest bond strengths (*p* < 0.0005). Cross-matching of acid and silane showed that acid had a statistically significant effect on bond strength. The H_3_PO_4_-Jeneric/Pentron silane group had the highest bond strength among all acid silane groups.	The Porcelain Primer group had the lowest bond strength, showing statistically significant differences to those of the Jeneric/Pentron groups (either phosphoric acid or HF acid etching) (*p* < 0.0005). Although acid might be more important than silane (*p* = 0.005) for bond strength, there were no statistically significant differences in bond strength among the other four etching-silane groups (phosphoric acid vs. HF acid; Ultradent vs. Jeneric/Pentron).
Durgesh et al., 2016 [49]	In vitro	A silane-based primer consisting of 1.0 vol-% of 3 acryloxypropyltrimethoxysilane (ACPS) in 95.0 vol-%/5.0 vol-% ethanol/water, with a ph of 4.5; experimental primer, a novel silane system consisting of 0.5 vol-% of a cross-linker silane monomer bis-1,2-(triethoxysilyl) ethane (BTSE) which was added to 1.0 vol-% of acps, corresponding to a final 1.5 vol-% of silanes.	Glazed ceramic porcelain fused to metal (PFM)	180	Two groups of 90 specimens, according to the primer used. Each group was further divided into three subgroups according to the surface treatment to be received, thus there were 6 study groups; three with 3-acryloxypropyltrimethoxysilane (ACPS) silane primer, namely 1a (pretreatment with hydrofluoric acid, HF), 1b (pretreatment with grit-blasting) and 1c (pretreatment with tribochemical silica-coating) and 3 with a novel silane system (ACPS + bis-1,2(triethoxysilyl) ethane (BTSE)) assigned as 2a (HF), 2b (grit-blast), and 2c (tribochemical silica coating).	NR	NR	SBST	The highest SBST at baseline (26.8 + 1.7 MPa) and after thermocycling (24.6 + 1.7 MPa) was observed in group 2c, and the lowest (9.6 + 1.5 MPa and 4.5 + 1.1 MPa) was found in G1a.	The application of experimental silane primer system on specimens pretreated with tribochemical silica-coating demonstrated increased adhesion of orthodontic brackets making it an excellent choice in orthodontic bonding for a relatively long term use.
Bavbek et al., 2014 [50]	In vitro	Air abrasion with 30-μm silica coated aluminum oxide (Al_2_O_3_) particles (cojet) for 20 s; air abrasion with 50-μm Al_2_O_3_ particles.	Monolithic zirconium oxide ceramic (mz).	120	Two types of MZ (BruxZir Solid Zirconia, n = 60; Prettau-Zirkon, n = 60) with two types of surface finish (glazed, n = 30 per group; polished, n = 30 per group) were tested after two surface conditioning methods: 1. air abrasion with 30-μm silica coated aluminum oxide (Al_2_O_3_) particles (CoJet), or 2. air abrasion with 50-μm Al_2_O_3_ particles.	The non-conditioned group acted as the control.	NR	SBST	Mean μSBST values (MPa) did not show a significant difference between the two brands of MZ (*p* > 0.05). In both glazed (44 ± 6.4) and polished (45.9 ± 4.8) groups, CoJet application showed the highest μSBST values (*p* < 0.001). The control group (34.4 ± 6) presented significantly better results compared to that of Al_2_O_3_ (30 ± 3.8) (*p* < 0.05) on glazed surfaces, but it was the opposite in the polished groups (control: 20.3 ± 4.7; Al_2_O_3_: 33.8 ± 4.7; *p* < 0.001).	Air abrasion with CoJet followed by the application of universal primer improved the μSBST (microshear bond strength) of orthodontic resin to both the polished and glazed monolithic zirconium oxide materials tested
Sandoval et al., 2020 [51]	In vitro	Hydrofluoric acid 10% + silane; sandblasting with aluminum oxide + silane; hydrofluoric acid 10% + Single Bond Universal; blasting with aluminum oxide + Single Bond Universal.	NR	60	G1: hydrofluoric acid + 10% silane; G2: blasting with aluminum oxide + silane; G3: hydrofluoric acid 10% + Single Bond Universal and G4: blasting with aluminum oxide + Single Bond Universal.	NR	Metallic	SBST	The average shear strengths were: G1 = 24.2 MPa; G2 = 21.3 MPa; G3 = G4 = 19.1 MPa to 14.2 MPa. There were differences between all groups (*p* < 0.05) except for G3 (*p* > 0.05).	Single Bond Universal treated with blasting aluminum oxide had the best performance, and promoted good shear strength, it caused less cohesive damage to the ceramic.
Najafi et al., 2014 [52]	In vitro	Roughened with a diamond bur and etched with hydrofluoric acid (HF) gel for 4 min; roughened with a bur and irradiated by a CO_2_ laser with a 2W power setting for 20 s; CO_2_ laser; sandblasted with 50 μm aluminum oxide for 20 s. Before bonding, the bracket silane was applied on the porcelain surfaces.	Feldspathic porcelain fused to metal.	48	Four groups: G1: Deglazed +HF group; G2: Deglazed +CO_2_ group; G3: CO_2_ group; G4: Sandblasted group. In the four groups, a silane coupling agent) was applied.	NR	Metallic	SBST	ANOVA revealed significant differences in SBS among the four groups (*p* < 0.001). G1 demonstrated significantly higher bond strength (13.13–2.47) when compared with the other groups. G2 showed higher bond strength (9.60–1.91) when compared with G4 (6.40–1.67) (*p* = 0.016).	Deglazing combined with HF etching produced the highest bond strength, but CO_2_ laser irradiation provided adequate bond strength and allowed for elimination of the HF step. Deglazing is not recommended as a preliminary step before CO_2_ laser conditioning.
Durgesh, 2020 [20]	In vitro	Grit-blasted with various distance (5 mm, 10 mm and 15 mm) with 1.0 vol. % 3 methacryloyloxypropyltrimethoxy-silane (ep1) or their blends with 0.5% (ep2), and 1.0 vol. % (ep3) 1, 2-bis-(triethoxysilyl) ethane (all in ethanol/water).	Zirconia	180	A total of 180 zirconia specimens were used for three test groups (n = 60), and then grit-blasted with various distance (5 mm, 10 mm and 15 mm). The grit-blasted specimens were allocated to three silanizations (n = 30): with 1.0 vol. % 3 methacryloyloxypropyltrimethoxysilane (EP1) or their blends with 0.5% (EP2), and 1.0 vol. % (EP3) 1, 2-bis-(triethoxysilyl) ethane (all in ethanol/water).	NR	NR	AST	ANOVA showed a significant influence of the grit-blasting distance, silane blend and artificial aging on the shear bond strength values (*p* < 0.05). The highest adhesion strengths were obtained for baseline specimens irrespective of the grit-blasting distance or the silane primer blend system used.	Grit-blasting at 10 mm and silane primer blend of 1.0 vol. % 3-MPS and 0.5 vol. % BTSE provided acceptable orthodontic bonding with least surface damage to zirconia surface. Adhesion strength values significantly decreased following thermo-cycling, irrespective of the grit-blasting distance and the silane primer blend system used.
García-Sanz et al., 2019 [53]	In vitro	Air particle abrasion (APA) with alumina particles (Al_2_O_3_) for 20 s; femtosecond Ti:sapphire laser for 12 min.	Zirconia	180	Five groups (n = 30) according to surface treatment: G1: air-particle abrasion (APA); G2: FS laser irradiation (300 mW output power, 60 μm inter-groove distance); G3: FS laser irradiation (200 mW, 100 μm); G4: FS laser irradiation (40 mW, 60 μm); G5: FS laser irradiation (200 mW, 60 μm).	G1- control group: No treatment applied (n = 30).	NR	SBST	SBST in groups 3 and 6 was significantly higher than the other groups (5.92 ± 1.12 MPa and 5.68 ± 0.94 MPa). No significant differences were found between groups 1, 2, 4, and 5 (3.87 ± 0.77 MPa, 4.25 ± 0.51 MPa, 3.74 ± 0.10 MPa, and 3.91 ± 0.53 MPa).	FS laser at 200 mW, 60 μm can be recommended as the ideal settings for treating zirconia surfaces, producing good SBST and more economical energy use.
Stella et al., 2015 [54]	In vitro	G1: 37% gel phosphoric acid etching for one minute + Silane application for one minute; G2: 37% liquid phosphoric acid etching for one minute+ Silane application for one minute; G3: 10% hydrofluoric acid etching for one minute; G4: 10% hydrofluoric acid etching for one minute + Silane application for one minute.	NR	52	Four experimental groups (n = 13) were set up according to the ceramic conditioning method: G1: 37% phosphoric acid etching followed by silane application; G2: 37% liquid phosphoric acid etching, no rinsing, followed by silane application; G3: 10% hydrofluoric acid etching alone. G4: 10% hydrofluoric acid etching followed by silane application.	NR	Metallic	SBST	The highest shear bond strength values were found in groups G3 and G4 (22.01 ± 2.15 MPa and 22.83 ± 3.32 Mpa, respectively), followed by G1 (16.42 ± 3.61 MPa) and G2 (9.29 ± 1.95 MPa).	Acceptable levels of bond strength for clinical use were reached by all methods tested; however, liquid phosphoric acid etching followed by silane application (G2) resulted in the least damage to the ceramic surface.
Epperson et al., 2021 [55]	Ex vivo	9.6% HF was for 4 min; 35% phosphoric acid (PA) with subsequent silanation; 50 μ aluminum oxide microetching (MIC)	Hybrid ceramics	60	G1: Lava (HF); G2: Lava (PA); G3 Lava (MIC); G1 Enamic (HF); G2 Enamic (PA); G3 Enamic (MIC).	Enamel control group (n = 10) (35% phosphoric acid for 30s and rinsed for 10 s. An adhesive primer, Transbond™ XT Primer was applied for 5 s and lightly air-thinned for 1 s.	Metallic	SBST	The SBST of all groups, except the HF Enamic^®^ group, were significantly lower than the mean SBS of the enamel control group (8.8 MPa). The mean shear bond strength values of Enamic^®^ were significantly higher than those of Lava™ Ultimate (*p*-values < 0.05).	Statistically, only Enamic^®^ treated with HF exhibited sufficient SBST when compared with the enamel control.
Al-Hity et al., 2012 [19]	In vitro	The influence of using different combinations of bracket, adhesive, and light- curing source on the tensile bond strength to porcelain Tensile tests were performed using: one ceramic bracket versus one metal bracket, two orthodontic composites; type bisphenol A-glycidyldimethacrylate and urethane dimethacrylate (UDMA), and four light- curing units with the same range of emission spectrum but various light intensities: three light- emitting diode (LED) units and one halogen-based unit.	Fluorapatite glass-ceramic-	160	160 porcelain samples were randomly divided into 16 equal groups. The porcelain surface was conditioned with 9% hydrofluoric acid before silane application. The composite was photo- polymerized for 40 s.	NR	Metallic and ceramic	TBST	The bond strength in all groups was sufficient to withstand orthodontic treatment (>6 MPa). There was no statistical difference between the adhesives, but comparing bracket × light interaction, it was significantly higher with the ceramic bracket. No significant differences were seen between the metal bracket groups, but for the ceramic bracket, the results were significantly higher with the LED light	No significant difference between adhesives’ composition related to the bonding strength on porcelain. Bonding strength of ceramic brackets on porcelain is significantly higher than metal bracket. Bonding strength of ceramic bracket is significantly higher when an LED LCU of high light intensity is used compared to halogen-based or LED LCU with low intensity.
Ghozy et al., 2020 [13]	In vitro	9.5% HF for 1 min; 37% PA gel for 1 min.	VITABLOCS Mark II, VITAENAMIC, and IPS e.max CAD.	120	120 CAD/CAM ceramic blocks in 12 groups were fabricated from three different CAD/CAM ceramic materials: VITABLOCS Mark II, VITAENAMIC, and IPS e.max CAD. Each ceramic material group was divided into two etching groups: 60 metal (BM) and ceramic brackets(CB) of the upper right central incisor were bonded to the HF-treated blocks. Another 60 metal and CBs were bonded to the PA treated blocks.	NR	Metallic and ceramic	SBST	There were no significant differences in SBS values between the three CAD/CAM ceramic materials. The HF-treated specimens exhibited significantly higher SBS values than the PA-treated specimens. Also, the SBS values of CBs were significantly higher than the BM.	The CAD/CAM ceramic type did not influence SBST; however, HF exhibited significantly higher SBST compared to PA.
Ramos et al., 2012 [56]	Ex vivo	G2: Diamond bur and processed with phosphoric acid 37% for 30 s; G3: Etching with HF 10% for 1 min; G4: etching with HF 10% for 1 min and application of 2 layers of silanization agent.	NR	40	n = 10 for each group. G2: fine diamond bur + orthophosphoric acid gel 37%; G3: HF 10%; G4: HF 10% + silane.	G1-control group: No surface treatment (n = 10).	Ceramic	SBST	There was a significant difference (*p* < 0.05) between the control group and all other groups. There was no significant difference (*p* < 0.05) between treated porcelain surface with diamond bur + orthophosphoric acid gel 37% (4.8 MPa) and HF 10% (6.1 MPa), but the group treated with HF 10% had clinically acceptable bond strength values. The group treated with HF 10% + silane (17.5 MPa) resulted in a statistically significant higher tensile bond strength (*p* < 0.05). In G4, 20% of the porcelain facets displayed damage.	Etching of the surface with HF 10% increased the bond strength values. Silane application was recommended to bond a ceramic bracket to the porcelain surface to achieve bond strengths that are clinically acceptable.
Mehmeti et al., 2018 [57]	In vitro	G1: 5% HF for 2 min; G2: 37% phosphoric acid for 2 min, and subsequently, silane.	Feldspar-based porcelain PFM.	48	Four groups (n = 12): G1: Metal bracket bonded after surface conditioning with 37 per cent phosphoric acid and silane; G2: Metal bracket bonded after surface conditioning with 5% HF and silane; G3: Ceramic bracket bonded after surface conditioning with 37% phosphoric acid and silane; G4: Ceramic bracket bonded after surface conditioning with 5% HF and silane.	NR	Metallic and ceramic	SBST	SBST values of the groups etched with HF and silane, compared to the groups etched with phosphoric acid and silane, are not significantly increased. However, ceramic brackets show significantly higher SBST values than metallic brackets.	Both types of ceramic surface conditioning procedures have similar features and provide strong enough SBST values to realize the orthodontic treatment. Also, the assumption that only the type of bracket significantly affects the SBST value can be accepted.
Baeshen, 2021 [58]	In vitro	G1: Er-YAG laser for about 30 s + S coupling agent for 30 s; G2: Photodynamic therapy (PDT) using methylene blue (MB) photosensitizer at a concentration of 100 mg/L; G3: 9.5% of HF acid for 60 s +S coupling agent was applied and air dried for 60 s; G4: 9.5% HF acid for 60 s + ultrasonic bath UB along with distilled water and air dried for 120 s + S; G5: Sandblasting with Al_2_O_3_ particles for 15 s; G6: SECP (Monobond etch and prime) for 60s followed by rinse for 20 s; G7: Er,Cr:YSGG for 60 s + S.	Lithium di silicate (LDC)	70	Seven groups according to ceramic surface conditioning. G1: surface treated with Er-YAG laser and saline (S); G2: PDT using MBP + S; G3: HF (Hydrofluoric acid) +saline, G4: HF (Hydrofluoric acid) +ultrasonic bath (UB) + S; G5: sand blasting the glass ceramic surface with 120 um Al_2_O_3_; G6: LDC surface conditioned with SECP (Etch and Prime); G7: Er,Cr:YSGG + S on was irradiated on LDC.	G3 HF + S (control).	Metallic	SBST	SBST values of G2 HF acid + S displayed highest bond durability (22.28 ± 1.09 MPa). Whereas, specimens in G4 surface treated with 120 μm Al_2_O_3_ displayed lowest SBST scores (11.81 ± 0.55 MPa) and these bond scores were comparable to PDT using MBP + S (12.54 ± 1.09 MPa) (*p* > 0.05). LDC surface treated by Er,Cr:YSGG + S (21.11 ± 3.85 MPa), HF + UB + S (19.28 ± 0.52 MPa) exhibited results comparable to HF acid + S (*p* > 0.05).	LDC conditioned with HF–S still remains as gold standard. Use of PDT for surface treatment of LDC and bonded to metallic bracket is not recommended as it results in decreased bond durability. Use of Er,Cr:YSGG-S and HF + UB + S has a potential to be used alternatively to HF–S for LDC conditioning.
Tahmasbi et al., 2020 [9]	In vitro	G1: universal adhesive (Scotchbond^TM^ Universal adhesive) 20 s, air spray 5 s, light cured 10 s 650 mW/cm^3^; G2: universal adhesive/silane 1min, air spray 30 s, Scotchbond Universal adhesive 20 s, air spray 5 s, light curinf 10 s; G3: conventional adhesive–two layers of Single Bond 2 conventional adhesive 20 s, air sprayed 5 s, light cured 10 s; G4: conventional adhesive/silane–silane 1 min, air spray 30 s, two layers of Single Bond 2 conventional adhesive 20 s, air spray 5 s, light cured 10 s.	Feldspathic porcelain	56	n = 14–universal adhesive; n = 14–universal adhesive/silane; n = 14–conventional adhesive; n = 14–conventional adhesive/silane.	NR	NR	SBST	The highest SBST was noted in the universal adhesive/silane group (12.7 MPa) followed by conventional adhesive/silane (11.9 MPa), conventional adhesive without silane (7.6 MPa), and universal adhesive without silane (4.4 MPa).	SBST of bracket to porcelain mainly depends on the use of silane rather than the type of adhesive. Both universal and conventional adhesives yield significantly higher SBST in the presence of silane compared to that in the absence of silane
Mehmeti et al., 2017 [59]	In vitro	Phosphoric acid 120 s, composite resin-based bonding system, T light cured 40 s using light-emitting diode.	All-zirconium ceramic	20	n = 10–metallic bracket; n = 10–ceramic polycrystalline bracket.	NR	Metallic and ceramic polycrystalline	SBST	Force necessary to debond metallic brackets (sum of 10 tests = 70,797 N) of the zirconium crowns were higher than those of ceramic brackets (sum of 10 tests = 59,770 N), with a significant difference.	Metallic brackets compared with ceramic polycrystalline brackets, seem to create stronger adhesion with all- zirconium surfaces due to their better base surface design or retention mode. Also, ceramic brackets show higher fragility during debonding.
Yassaei et al., 2013 [60]	In vitro	G1: 9.6% HF; G2, 3 and 4: Er:YAG lasers of 1.6, 2, and 3.2 W.	Porcelain	100	Four groups: G1: n = 25–HF; G2: n = 25–Er:YAG lasers of 1.6; G3: n = 25–Er:YAG lasers of 2; G4: n = 25–Er:YAG lasers of 3.2	NR	Metallic	SBST	The mean shear bond strength in the laser group with power of 1.6 W (7.88 MPa) was more than that of the HF (7.4 MPa), 2-W power (7.52 MPa), and 3.2-W power (7.45 MPa) groups, but this difference was not statistically significant.	Er:YAG laser can be a suitable method for bonding of orthodontic brackets to porcelain surfaces.
Gardiner et al., 2019 [5]	In vitro	Hydrofluoric acid etch	Zirconia; lithium disilicate (IPS e.max); lithium silicate infused with zirconia (CELTRA DUO)	60	Zirconia (n = 20): 9.6%HF+silane (n = 10), silane (n = 10); IPS e.max (n = 20): 9.6% HF+silane (n = 10), silane (n = 10); CELTRA DUO (n = 20): 9.6%HF + silane (n = 10), silane (n = 10).	Enamel (n = 10): 35% PA etch	Metallic	SBST	SBST of the lithium silicate infused with zirconia groups were significantly less than the chemically pre-treated lithium disilicate group, however both materials, when chemical pre- treatment protocol was used, were not statistically different than the enamel control.	Orthodontic bonding to lithium silicate infused with zirconia yielded a weaker shear bond strength than bonding to traditional lithium disilicate, however, when the surface was pre- treated with hydrofluoric acid etch it provides a bond strength that is within an acceptable clinical range.
Golshah et al., 2018 [4]	In vitro	10% HF acid 2 min and the following bonding protocols: G1: Transbond XT bonding agent cured 10 s; G2: silane plus Transbond XT bonding agent cured 10 s; G3: silane plus universal adhesive (G-Premio bond) cured 10 s; G4: universal adhesive cured 10 s.	Glazed feldspathic porcelain	40	G1: Transbond XT bonding agent (n = 10); G2: silane plus Transbond XT bonding agent (n = 10); G3: silane plus universal adhesive (G-Premio bond) (n = 10); G4: universal adhesive (n = 10).	NR	Metallic	SBST	The highest and the lowest SBST values were noted in groups silane plus universal adhesive (17.06 ± 2.58 MPa) and universal adhesive (9.85 ± 4.76 MPa), respectively. Type of adhesive had no significant effect on SBST (*p* = 0.611). However, the effect of application of silane on SBST was significant (*p* = 0.000). Groups subjected to the application of silane showed higher SBST values than others.	Universal adhesive and Transbond XT were not significantly different in SBST. However, application of silane significantly increased the bond strength.
Hosseini et al., 2013 [61]	In vitro	0.75-, 1-, 1.25-, 1.5- and 2-W neodymium-doped yttrium aluminum garnet (Nd:YAG) laser 10 s; hydrofluoric acid 9,6% 4 min	Glazed porcelain	72	n = 12–HF; n = 12–0.75-Nd:YAG laser; n = 12–1-Nd:YAG laser; n = 12–1.25-Nd:YAG laser; n = 12–1.5-Nd:YAG laser; n = 12–2-Nd:YAG laser.	NR	Metallic	SBST	The mean ± SD of the shear bond strength in the laser group 0.75, 1, 1.25, 1.5, and 2 W and HF group was 2.2 ± 0.9, 4.2 ± 1.1, 4.9 ± 2.4, 7 ± 1.7, 9.6 ± 2.7, and 9.4 ± 2.5, respectively. Together with the increased power of laser, the mean shear bond strength was increased continuously and no significant differences were found between the HF group and the laser groups with power of 1.5 or 2 W.	1.5 and 2 W powers of Nd:YAG laser can be used as an alternative method for porcelain etching.
Naseh et al., 2018 [10]	In vitro	9.6% hydrofluoric acid and divided into two groups: silane, air-dried, Transbond XT primer light-cured; Assure Plus, air-dried.	Feldspathic; lithium disilicate	40	n = 10–feldspathic with Assure Plus; n = 10–IPS E-max with Assure Plus	n = 10–feldspathic with silane+Transbon; n = 10–IPS E-max with silane+Transbond.	Metallic	SBST	Bracket bond to lithium disilicate by Assure Plus was significantly stronger than that to Feldspathic porcelain (*p* = 0.041).	Assure Plus provided high bond strength between ceramic and brackets and minimized damage to lithium disilicate ceramic during debonding. Assure Plus is recommended for use in orthodontic treatment of adults with ceramic restorations.
Cevik et al., 2018 [17]	In vitro	G1: 37% phosphoric acid 4 min; G2: 9.6% hydrofluoric acid 3 min; G3: grinding with diamond burs 20 μm with a high-speed handpiece 30s in wet conditions; G4: Nd:YAG laser 15 Hz 1 W 30 s with pulse duration range was 300 μs; G5: Airborne-particle abrasion 50 μm alumina (Al_2_O_3_) particles 2.5 bars of pressure 10 s at a direction perpendicular to the surface with the distance of 10 mm.	Feldspathic porcelain	60	G1: 37% phosphoric acid (n = 10); G2: 9.4% hydrofluoric acid (n = 10); G3: grinding with diamond burs (n = 10); G4: Nd:YAG laser (n = 10); G5: Airborne-particle abrasion (n = 10).	Without surface treatment (n = 10).	Ceramic	SBST	Using G5 specimens resulted in the highest shear bond strength value of 8.58 MPa for feldspathic porcelain. However, the other specimens showed lower values: G3 (6.51 MPa), G4 (3.37 MPa), G2 (2.71 MPa), G1 (1.17 MPa), and control group (0.93 MPa).	Airborne-particle abrasion and grinding can be used as surface treatment techniques on the porcelain surface for a durable bond strength. Hydrofluoric acid and phosphoric acid etching methods were not convenient as surface treatment methods for the feldspathic porcelain.
Juntavee et al., 2018 [7]	In vitro	Er-YAG laser power 200 mJ, 10 W, 20 Hz, 10 s-pulse length for 20 s; Etching with 9.5% HF acid gel 5 s (E5) or 15 s (E15).	Machined ceramic specimens: Empress^®^ CAD, and e.max^®^ CAD; Ceramic veneering metal (d.Sign^®^ porcelain (1.27 mm thickness) over d.Sign^®^ 10 metal (0.23 mm thickness)).	45	n = 15–Er-YAG laser; n = 15–HF 5 s; n = 15–HF 15 s.	NR	Ceramic	SBST	Significant differences in bond strength among groups were found related to surface treatment (*p* < 0.05), but not significant difference upon type of ceramics (*p* > 0.05). E15 provided higher bond strength than Er-YAG laser and E5 (*p* < 0.05).	Bond strength was affected by surface treatment. Both Er-YAG laser and E15 treated surface provided higher bond strength than E5. Considering possibly inducing defect on ceramic surface, Er-YAG laser seems to provide better favorable surface preparation than others. Treated ceramic surface with Er-YAG prior to bracket bonding is recommended.
Sabuncuoglu et al., 2016 [62]	In vitro	G1: Cylindrical diamond bur rotate at 40,000 rpm 3 s; G2: 37% Orthosphoric acid 2 min; G3: 9,6% HF 2 min; G4: SB with 50 μm aluminum oxide at 60 psi for 3 s at a distance of 10 mm; G5: SB+HF; G6: Nd:YAG laser wavelength 1064nm (300 μm fiber), 2 W power and frequency of 10 Hz for 10 s in a pulse mode (100 μs) using a sweeping motion at approximately 2 mm distance; G7: Er:YAG laser 2 W, 10 Hz, 10 s, 2 mm.	Feldspathic	70	G1: Diamond bur (n = 10); G2: Orthosphoric acid (n = 10); G3: Hydrofluoric acid (n = 10); G4: Sandblasted with aluminum oxide (n = 10); G5: SB+HF (n = 10); G6: Nd:YAG laser (n = 10); G7: Er:YAG laser (n = 10).	NR	NR	SBST	The highest SBST values were observed for SB + HF, with no significant difference between SB+HF and HF. SBST values for Diamond bur were significantly lower than those of all other groups tested.	Diamond bur alone is unable to sufficiently etch porcelain surfaces for bracket bonding. SB+HF results in a significantly higher shear-bond strength than HF or SB alone. Nd:YAG or Er:YAG laser was found to be more effective and less time-consuming than both HF acid and SB.
Aksakalli et al., 2015 [63]	Ex vivo	G1: SB with alumina particles 50 μm, at 65–70 psi, 10 s, 10 mm (SB); G2: 9.6% HF 4 min; G3: Er: YAG irradiation from 1 mm distance, 2 W, 10 Hz, 200 mJ, 100-μs pulse length, energy density of 25.31 J/cm^2^ for 10 s.	Porcelain laminate veneer	39	G1: SB (n = 13); G2: HF (n = 13); G3: Er:YAG (n = 13).	NR	NR	SBST	The highest shear bond strength values were obtained with group HF (10.8 ± 3.8 MPa) and group ER (9.3 ±1.5 MPa), whereas group SB revealed the lowest values. The sandblasting method did not demonstrate any ideal bond strength values; however, the 9.6% hydrofluoric acid etching and Er: YAG laser did.	The Er: YAG laser can be selected for bonding brackets to porcelain surfaces with acceptable bond strength and minimal surface damage as compared to the other methods.
Lestrade et al., 2021 [64]	In vitro/Ex vivo	G1: Bond enhancer (Assure, Reliance, IL, USA); G2: Green stone at 25.000 rpm; G3: Diamond bur at 25.000 rpm; G4: SB 25 μm aluminum oxide particles, distance 10 mm,10 s; SB with Rocatec (3 M ESPE, MN, USA) 100 μm aluminum oxide particles treated with silicon dioxide.	Lithium disilicate	70	G1: Porc-Etch, 9.6% HF, Porcelain Conditioner, silane, bond enhancer (Assure, Reliance, IL, USA) (n = 10); G2: green stone (n = 10); G3: diamond burr (n = 10); G4: SB (n = 10); G5: SB with Rocatec (n = 10).	n = 10–HF + silane; n = 10–teeth with 37% phosphoric acid + self-etching + primer + adhesive.	Metallic	SBST	No significant differences were found in SBST values, with the exception of surface roughening with a green stone prior to HF and silane treatment. This protocol yielded slightly higher bond strength which was statistically significant	The present in-vitro study found that SBST values for ceramic pretreatment all fell within an acceptable clinical range and similar to the bond strength of enamel. No significant differences were found in the SBST values, with the exception of roughening with a green stone prior to HF and silane treatment, which yielded slightly higher bond strength.
Poosti et al., 2012 [65]	In vitro	Tungstem carbide burrs; 9.6% Hydrofluoric acid 4 min; Er:YAG; Neodymium-doped yttrium aluminum garnet laser (Nd: YAG) 10 s.	Glazed porcelain	100	G1: tungsten carbide burs (n = 20); G2: tungsten carbide burs + 9.6% hydrofluoric acid (n = 20); G3: 0.8-W Nd:YAG laser (n = 20); G4: 2W Er:YAG laser (n = 20); G5: 3-W Er:YAG laser (n = 20).	NR	Metallic	SBST	Although Tukey’s test showed SBST in tungsten carbide burs+ 9.6% hydrofluoric acid and Nd:YAG laser were significantly higher than the other groups, they did not differ with each other significantly (*p* > 0.05). The results revealed that SBST of 9.6% hydroflouric acid and Nd:YAG Laser was in an acceptable range for orthodontic treatment.	Nd:YAG laser was shown to be an acceptable substitute for hydrofluoric acid while Er:YAG laser with the mentioned power and duration was not a suitable option.
AlShahrani et al., 2019 [66]	In vitro	G1: 9.6% Hydrofluoric acid+ S 60 s; G2: Er,Cr:YSGG laser 2 mm, 4.5 W, 30 Hz, 60 s; G3: Fractional carbon dioxide (CO_2_) laser 10 W, 200 Hz, 3 mm, 60s, pulse duration 1.75 ms; G4: SB Al_2_O_3_ 10 mm, 2.8 MPa, 20 s; G5: Monobond etch & prime, Ivoclar Vivadent, Schaan, Liechtenstein.	Lithium disilicate	50	G2: Er,Cr:YSGG laser+ S (n = 10); G3: CO_2_ laser + S (n = 10); G4: SB (n = 10); G5: Self-Etch Glass Ceramic Primer (n = 10).	G1: HF+ S (n = 10).	Metallic and ceramic polycrystalline	SBST	The highest SBST values were presented by HF + S (21.08 ± 1.06). The lowest SBST values were displayed by Al_2_O_3_ (12.61 ± 0.45). SBST of samples conditioned with self-etch glass ceramic primer showed significant difference amongst all experimental groups (16.76 ± 0.81).	Lithium disilicate ceramics photosensitized with CO_2_ and Er,Cr:YSGG has a potential to be recommended in clinical settings alternate to HF+S when bonded to metallic bracket.
May et al., 2015 [67]	In vitro	10% HF 20 s; aluminum oxide blasting 15 s, pressure at 80 psi, 5 mm; 35% phosphoric acid 30 s; CoJet blasting; S.	Eris ceramic; d. Sign ceramic	120	n = 60–Eris Ceramic; n = 15 -10% HF; n = 15–10% HF + S; n = 15 -aluminum oxide blasting + 35% phosphoric acid + S; n = 15 -CoJet blasting + 35% phosphoric acid + S); n = 60–d.Sign Ceramic; n = 15 -10% HF; n = 15–10% HF + S; n = 15 -aluminum oxide blasting + 35% phosphoric acid + S; n = 15 -CoJet blasting + 35% phosphoric acid + S).	NR	Metallic	SBST	There were statistically significant differences among the ceramics (*p* = 0.01) and surface treatments (*p* = 0.0001), but it did not show interaction among them (*p* = 0.14).	The tested ceramics performed similarly in terms of bond strength; the use of S after HF was responsible for the increase of bond strength values; HF+ S, as well as aluminum oxide +phosphoric acid+S provided significantly higher bond strength values to metallic brackets; the CoJet system did not result in significantly higher values than those observed for aluminum oxide blasting, becoming similar to the groups treated with HF without S; aluminum oxide blasting followed by phosphoric acid etching and S presented results similar to the treatment with HF + S.
Alqerban, 2021 [68]	In vitro	G2: S; G3: HF 9.5% 60 s; G4: SB 50 um Al_2_O_3_ 1 mm, 2.8 atm, 15 s; G5: Self-etch ceramic primer (Monobond etch & prime, Ivoclar Vivadent, Schaan, Liechtenstein) (SECP) 60 s; G6: Er,Cr:YSGG laser 4.5 W, 30 Hz, 1 mm.	Lithium disilicate	90	G2: S 30 s (n = 15); G3: HF + UB + S 20 s (n = 15); G4: SB (n = 15); G5: SECP (n = 15); G6: Er,Cr:YSGG laser + S 20s (n = 15).	G1: HF + S 20s (n = 15).	Metallic	SBST	The highest SBST values were observed in HF+ UB + S (18.21 ± 1.241) and the lowest SBST values IN S only (5.21 ± 0.23). Specimens surface conditioned with HF+ S (17.85 ± 1.25), HF+ UB + S (18.21 ± 1.241) and Er,Cr:YSGG laser+ S (17.09 ± 1.114) unveiled comparable SBST values (*p* > 0.05).	Lithium disilicate conditioned with Er,Cr:YSGG laser has a potential to be used in clinical settings alternate to HF.
Elsaka, 2016 [69]	In vitro	9% HF 1 min; 37% H_3_PO_4_ 1 min; Diamond ceramic grinding bur (VOCO, Cuxhaven, Germany) 6000–10,000 rpm; Silica coating using CoJet system (CJ).	Vita Enamic (VE) CAD/CAM hybrid ceramic	240	n = 120–ceramic bracket (n = 30-HF; n = 30-H_3_PO_4_; n = 30–diamond ceramic grinding bur; n = 30-CJ). n = 120–metal bracket (n = 30-HF; n = 30-H_3_PO_4_; n = 30-diamond ceramic grinding bur; n = 30–CJ).	NR	Ceramic and metal brackets	SBST	SBST was significantly affected by the type of bracket and by type of treatment (*p* < 0.001). Specimens treated with CJ presented with significantly higher SBST compared to other groups (*p* < 0.05). Improvements in SBST values (MPa) were found in the following order: CJ [ HF [ Bur [H_3_PO_4_. Ceramic bracket showed higher SBST compared to metal bracket.	Surface treatment of VE CAD/CAM hybrid ceramic with silica coating enhanced the adhesion with ceramic and metal brackets. Ceramic bracket provided higher bond strength com- pared to metal bracket.
Falkensammer et al., 2012 [70]	In vitro	5% HF 60/30 s, 9.6% buffered HF 9.6%, 60/30 s; SB Al_2_O_3_/SiO_2_ particles.	Metal- and all-ceramic veneering: feldspathic; leucite; leucite-free; fluorapatite.	960	The four types of ceramic were allocated to each of the six conditioning groups, resulting in 24 subgroups of 40 brackets each.	NR	NR	SBST	HF 5% or SB resulted in significantly (*p* < 0.001) higher bond strengths (mean values: 34.11 and 32.86 MPa, respectively) than with HF 9.6% (mean value: 12.49 MPa). Etching time or SB particles had no statistical (*p* > 0.001) influence on bond strength.	Different conditioning procedures have an effect on ceramic microstructures and bracket adhesion. High SBST (29.74–36.80 MPa) were found for all ceramic surfaces when HF 5% or SB, indicating a higher risk of ceramic fracture. The HF 9.6% appeared to have a minor conditioning effect, resulting in a lower SBST (9.34–15.92 MPa), but fewer ceramic fractures. A short etching time (30 s) was as effective as standard etching (60 s). SB SiO_2_ showed no advantage as compared with SB AL_2_O_3_.
Kim et al., 2017 [71]	In vitro	SB Al_2_O_3_ and CO (Colet TM); S; Zirconia Prime Plus (ZPP) and SBU.	Zirconia	120	n = 10–Al_2_O_3_ + S-T; n = 10–Al_2_O_3_ + S-N; n = 10–Al_2_O_3_ + ZPP-T; n = 10–Al_2_O_3_ + ZPP-N; n = 10–Al_2_O_3_ + SBU-T; n = 10–Al_2_O_3_ + SBU-N; n = 10–CO + S-T; n = 10–CO + S-N; n = 10–CO + ZPP-T; n = 10–CO + ZPP-N; n = 10–CO + SBU-T; n = 10–CO + SBU-N.	NR	Metallic	SBST	CO-SBU had the highest bond strength after T. CO-S significantly higher SBST than Al_2_O_3_-S. CO-ZPP lower bond strength than Al_2_O_3_-ZPP before thermocycling, but the SBST increased after T.	CO-SBU showed the highest shear bond strength. Sandblasting with either AL or CO improved the mechanical bonding by increasing the surface area, and all primer groups showed clinically acceptable increase of SBST for orthodontic treatment.
Alaqeel, 2020 [72]	In vitro	Heat-treatment	Lithium disilicate glass-ceramic	120	n = 60–heat treated specimens (n = 15–neutralized, bonded with resin based cement; n = 15–neutralized, bonded with water based cement; n = 15–non-neutralized, bonded with resin based cement; n = 15–non-neutralized, bonded with water based cement).	n = 60–non-heat treated specimens (n = 15–neutralized, bonded with resin based cement; n = 15–neutralized, bonded with water based cement; n = 15–non-neutralized, bonded with resin based cement; n = 15–non-neutralized, bonded with water based cement).	NR	SBST	The heat-treated showed statistically significant higher bond strength in all the sub- groups, and the acid-neutralized samples showed higher bond strength using both types of cement; however, the increase was statistically significant only in resin-based cement-bonded samples. Resin-based cement-bonded samples showed higher bond strength than water-based cement-bonded samples.	Pre-etching heat treatment and post-etching acid neutralization of the cementing surface of lithium disilicate glass-ceramic significantly improve the initial bond strength to orthodontic brackets.
Guida et al., 2019 [73]	In vitro	10% HF 60s; S 3 min; HF + S; MDP–The adhesive system (Ambar, FGM, Joinville, Brazil—primer and adhesive combined in one bottle) containing MDP (10-methacryloyloxydecyl dihydrogen phosphate) applied in two layers. The first drop of adhesive was vigorously brushed on the bonding surface for 10 s, air-thinned before the second layer of adhesive was applied for 10 s, air-thinned, and light cured for 10 s.	Lithium disilicate-based glass–ceramic.	240	n = 20–BM-HF; n = 20–BM-S; n = 20–BM- HF + S; n = 20–BM-MDP; n = 20–BCp-HF; n = 20–BCp-S; n = 20–BCp-HF + S; n = 20–BCp–MDP; n = 20–BCm-HF; n = 20–BCm-S; n = 20–BCm-HF + S; n = 20–BCm-MDP.	BM—Stainless steel, metal bracket (Abzil, 3M Brazil, São Jose do Rio Preto, SP, Brazil) with a traditional mesh for mechanical retention	Metallic (BM) and ceramic brackets (monocrystalline (BCm) and polycrystalline (BCp).	SBST	BCm with HFS or HF showed the highest median σ values, 10.5 MPa and 8.5 MPa respectively. In contrast, the BCp with MDP showed the lowest median σ value (0.8 MPa), which was not statistically different from other MDP-treated groups.	The failure mode was governed by the glass–ceramic surface treatment, not by the bracket type. Quantitative (σ values) and qualitative (fracture mode) data suggested a minimum of 5 MPa for brackets bonded to glass–ceramic, which is the lower critical limit bond strength for a comprehensive orthodontic treatment.
Martalia et al., 2020 [74]	In vitro	9% HF; Silane Ultradent; Ortho Solo bonding and Grengloo; adhesive bracket; Single Bond Universal 3 M ESPE.	Porcelain veneers	28	n = 7–9% HF 90 s, aquades 5 s, air spray 5 s, Silane, air spray 60 s, Ortho Solo, Grenglo adhesive, light-curing 20 s; n = 7–9% HF 90 s, aquades 5 s, air spray 5 s, Single Bond, light-curing 10 s, Grenglo adhesive, light- curing for 20 s; n = 7–9% HF 90 s, aquades 5 s, air spray 5 s, Ortho Solo, Grenglo adhesive, light- curing for 20 s.	n = 7–Single Bond, light-curing 10 s, apply Grenglo adhesive to bracket mess and the last step do light-curing for 20 s.	Metallic	SBST	The shear bond strengths between groups were significantly different (*p* < 0.05). The greatest bracket shear bond strength and lowest porcelain surface roughness were found in hydrofluoric acid, silane, bonding, and adhesive.	Silane applied separately from bonding and acid has great shear bond strength and low porcelain surface roughness.
Jivanescu et al., 2014 [75]	In vitro	Relyx U200 dual cure resin cement; Blugloo light cure composite	Porcelain-fused-to-metal	16	n = 8-Relyx U200 dual cure resin cement; n = 8-Blugloo light cure composite.	NR	Metallic	TBST	No statistically significant differences among the two cements in terms of tensile bond strength.	Both dental materials may be recommended for orthodontic bracket bonding to ceramic surfaces, with equally successful results. However, further testing on an increased number of specimens may be considered for more accurate data.
Park et al., 2013 [76]	In vitro	37% Phosphoric acid 1 min, wash, dry with electric light 5 min, light curing 40 s, Transbond 37 °C of water bath for 24 h. Laser irradiation–Er:YAG laser 140 μm of the wave length and 20% of air water ratio, 20 Hz per second, 2 W, 1 mm distance, 20 s irradiation, dry 5 s, Transbond 40 s enlightening 37 °C of water bath for 24 h.	Zirconia and Ceramic	150	n = 10-Metal with phosphoric acid; n = 10-Metal with laser acid; n = 10-Metal with laser + phosphoric acid; n = 10-Ceramic with phosphoric acid; n = 10-Ceramic with laser acid; n = 10-Ceramic with laser + phosphoric acid; n = 10-Zirconia with phosphoric acid; n = 10-Zirconia with laser acid; n = 10-Zirconia with laser + phosphoric acid.	n = 10-Tooth with Phosphoric acid; n = 10-Tooth with Laser acid; n = 10-Tooth with Laser + phosphoric acid.	NR	SBST	Changed as the most in ceramic in laser irradiation. Bonding strength according to the etching method was the most in laser irradiation and acid etching in ceramic and in zirconia.	Ceramic crown with acid treatment was recommended because of relatively high in bonding strength.
Hellak et al., 2016 [77]	In vitro/Ex vivo	Self-etching no-mix adhesives (iBond^TM^ and Scotchbond^TM^); Total etch system Transbond XT^TM^.	Glass-ceramic veneering (IPS e.maxTM Press, IPS e-max ZirCAD for inLabTM was used as high-strength zirconia and VITAblocsTM Mark II, C2II4 for CERECTM/inLab (VITA Zahnfabrik, Bad Sackingen, Germany) was used as a monochromatic feldspathic ceramic.	270	n = 240 divided into eight restorative surface groups (n = 30), of which Glass-ceramic veneering (n = 90) and all of the surfaces were divided into three subgroups with different adhesives (n = 10).	n = 30-Human enamel with Transbond XT primer; n = 30-Human enamel with iBond. n = 30-Human enamel with Scothchbond Universal	Metallic	SBST	Significant differences in SBST were found between the control group and experimental groups.	Transbond XT showed the highest SBST on human enamel. Scotchbond Universal on average provides the best bonding on all other types of surface (metal, composite, and porcelain), with no need for additional primers. It might therefore be helpful for simplifying bonding in orthodontic procedures on restorative materials in patients.
Lee et al., 2015 [32]	In vitro	G1: SB 50 µm, 5 s at pressure of 40 psi, 5 mm; G2: 9% HF acid 4 min; G3: porcelain primer (PP) thin coat; G4: zirconia primer (ZP) thin coat.	Zirconia	40	G1: nonglazed zirconia treated with SB + ZP (n = 10); G2: glazed zirconia treated with SB + etching + ZP (n = 10); G3: glazed zirconia treated with SB + etching + PP (n = 10); G4: glazed zirconia treated with SB + etching + ZP + PP (n = 10).	NR	Metallic	SBST	Group G2 showed significantly lower shear bond strength than did the other groups. No statistically significant differences were found among groups G1, G3, and G4.	Porcelain primer is the more appropriate choice for bonding a metal bracket to the surface of a full-contour glazed zirconia crown with resin cement.

ACPS—3-acryloxypropyltrimethoxysilane; Al2O3—aluminium oxide; ANOVA—one-way analysis of variance; APA—Air-particle abrasion; ARI—adhesive Remnant Index; AST—adhesion strength test; atm—standard atmosphere; BCm—monocrystalline brackets; BCp—polycrystalline brackets; BIS-EMA—bisphenol A diglycidyl methacrylate ethoxylated; BIS-GMA—bisphenol A-glycidyl methacrylate; BM—metallic brackets; BTSE—bis-1,2-(triethoxysilyl) ethane; CB—ceramic brackets; Cj—CoJet system; CO—Colet TM; CO2—carbon dioxide; CSBS—cyclic shear bond strength; DB—deglazing using diamond bur; df—degrees of freedom; E15—hydrofluoric acid 15s; E5—hydrofluoric acid 5s; Er: CrYSGG—erbium, chromium:yttrium-scandium-gallium-garnet; Er:YAG—Erbium:yttrium-aluminum-garnet; FC—feldspathic ceramic; G—group; H3PO4—orthophosphoric acid; HF—hydrofluoric acid; Hz—hertz; IP—IPS e.max CAD; LDC—lithium di silicate; Led—light—emitting diode; LU—lava ultimate; MB—methylene blue; MDP—10-methacryloyloxydecyl dihydrogen phosphate; MEP—monobond etch & prime; MIC—aluminum oxide microetching; min—minute(s); mj—millijoule; mm—millimeter; MPa—megapascal pressure unit; mz—Monolithic zirconium oxide ceramic; Nd:YAG—neodymium-doped yttrium aluminium garnet; np—no-primer; NR—not reported; *p*—*p*-value; PA—phosphoric acid; PDT—photodynamic therapy; PFM—porcelain fused to metal; PP—porcelain primer; psi—pounds of force per square inch of área; rpm—revolutions per minute; s—second(s); S—silane; S@SB2—adper single bond 2; S@SBU—silane + single bond universal; SB—sandblasting; SB2—single bond 2; SBST—shear bond strength test; SBU—single bond universal; sd—standard deviation; SECP—monobond etch and prime; SiO2—silicon dioxide; sp—short pulse; ssp—super short pulse; T—thermocycled; TBST—tensile bond strength test; TEGDMA—triethylene glycol dimethacrylate; TS—tribochemical silica coating UB—ultrasonic bath; UDMA—bisphenol A-glycidyldimethacrylate and urethane dimethacrylate; VE—Vita Enamic; VM—VITA Mark II; ZP—zirconia primer; ZPP—zirconia prime plus.

## Data Availability

The data presented in this study are available on request from the corresponding author.

## References

[1] Alzainal A.H., Majud A.S., Al-Ani A.M., Mageet A.O. (2020). Orthodontic Bonding: Review of the Literature. Int. J. Dent..

[2] González-Serrano C., Phark J.-H., Fuentes M.V., Albaladejo A., Sánchez-Monescillo A., Duarte S., Ceballos L. (2021). Effect of a single-component ceramic conditioner on shear bond strength of precoated brackets to different CAD/CAM materials. Clin. Oral Investig..

[3] Lopes G.V., Correr-Sobrinho L., Correr A.B., de Godoi A.P.T., Vedovello S.A.S., de Menezes C.C. (2020). Light Activation and Thermocycling Methods on the Shear Bond Strength of Brackets Bonded to Porcelain Surfaces. Braz. Dent. J..

[4] Golshah A., Mohamadi N., Rahimi F., Pouyanfar H., Tabaii E., Imani M. (2018). Shear Bond Strength of Metal Brackets to Porcelain Using a Universal Adhesive. Med. Arch..

[5] Gardiner R., Ballard R., Yu Q., Kee E., Xu X., Armbruster P. (2019). Shear bond strength of orthodontic brackets bonded to a new all-ceramic crown composed of lithium silicate infused with zirconia: An in vitro comparative study. Int. Orthod..

[6] Durgesh B.H., Alhijji S., Hashem M.I., Al Kheraif A.A., Durgesh P., Elsharawy M., Vallittu P.K. (2016). Influence of tooth brushing on adhesion strength of orthodontic brackets bonded to porcelain. Biomed. Mater. Eng..

[7] Juntavee N., Juntavee A., Wongnara K., Klomklorm P., Khechonnan R. (2018). Shear bond strength of ceramic bracket bonded to different surface-treated ceramic materials. J. Clin. Exp. Dent..

[8] Mehmeti B., Kelmendi J., Iiljazi-Shahiqi D., Azizi B., Jakovljevic S., Haliti F., Anić-Milošević S. (2019). Comparison of Shear Bond Strength Orthodontic Brackets Bonded to Zirconia and Lithium Disilicate Crowns. Acta Stomatol. Croat..

[9] Tahmasbi S., Shiri A., Badiee M. (2020). Shear bond strength of orthodontic brackets to porcelain surface using universal adhesive compared to conventional method. Dent. Res. J. (Isfahan.).

[10] Naseh R., Afshari M., Shafiei F., Rahnamoon N. (2018). Shear bond strength of metal brackets to ceramic surfaces using a universal bonding resin. J. Clin. Exp. Dent..

[11] Pinho M., Manso M.C., Almeida R.F., Martin C., Carvalho Ó., Henriques B., Silva F., Pinhão Ferreira A., Souza J.C.M. (2020). Bond Strength of Metallic or Ceramic Orthodontic Brackets to Enamel, Acrylic, or Porcelain Surfaces. Materials.

[12] Kaya Y., Unalan Degirmenci B., Degirmenci A. (2019). Comparison of the Shear Bond Strength of Metal Orthodontic Brackets Bonded to Long-term Water-aged and Fresh Porcelain and Composite Surfaces. Turkish J. Orthod..

[13] Ghozy E.A., Shamaa M.S., El-Bialy A.A. (2020). In vitro testing of shear bond strength of orthodontic brackets bonded to different novel CAD/CAM ceramics. J. Dent. Res. Dent. Clin. Dent. Prospects.

[14] Kara M., Demir O., Dogru M. (2020). Bond Strength of Metal and Ceramic Brackets on Resin Nanoceramic Material With Different Surface Treatments. Turkish J. Orthod..

[15] Juntavee P., Kumchai H., Juntavee N., Nathanson D. (2020). Effect of Ceramic Surface Treatment and Adhesive Systems on Bond Strength of Metallic Brackets. Int. J. Dent..

[16] Alavi S., Samie S., Raji S.A.H. (2021). Comparison of lithium disilicate–reinforced glass ceramic surface treatment with hydrofluoric acid, Nd:YAG, and CO2 lasers on shear bond strength of metal brackets. Clin. Oral Investig..

[17] Cevik P., Eraslan O., Eser K., Tekeli S. (2018). Shear bond strength of ceramic brackets bonded to surface-treated feldspathic porcelain after thermocycling. Int. J. Artif. Organs.

[18] Faggion C.M. (2012). Guidelines for Reporting Pre-clinical In Vitro Studies on Dental Materials. J. Evid. Based Dent. Pract..

[19] Al-Hity R., Gustin M.-P., Bridel N., Morgon L., Grosgogeat B. (2012). In vitro orthodontic bracket bonding to porcelain. Eur. J. Orthod..

[20] Durgesh B.H. (2020). Experimental silane primer and grit-blasting distance in orthodontic bonding of zirconia surfaces. Ceram.-Silikaty.

[21] Mohammed M.A., Bhuminathan S. (2019). A Comparative Evaluation of Shear Bond Strength of Orthodontic Brackets Bonded to Porcelain Fused Metal Crowns Treated with Different Surface Conditioning Techniques―An Invitro Study. Indian J. Public Health Res. Dev..

[22] Dilber E., Aglarcı C., Akın M., Özcan M. (2016). Adhesion of metal brackets to glassy matrix and hybrid CAD/CAM materials after different physico-chemical surface conditioning. J. Adhes. Sci. Technol..

[23] Miersch S., König A., Mehlhorn S., Fuchs F., Hahnel S., Rauch A. (2020). Adhesive luting of orthodontic devices to silica-based ceramic crowns—Comparison of shear bond strength and surface properties. Clin. Oral Investig..

[24] Kurt İ., Çehreli Z.C., Özçırpıcı A.A., Şar Ç. (2019). Biomechanical evaluation between orthodontic attachment and three different materials after various surface treatments: A three-dimensional optical profilometry analysis. Angle Orthod..

[25] Zhang Z., Qian Y., Yang Y., Feng Q., Shen G. (2016). Bond strength of metal brackets bonded to a silica-based ceramic with light-cured adhesive. J. Orofac. Orthop./Fortschritte der Kieferorthopädie.

[26] Recen D., Yıldırım B., Othman E., Çömlekoğlu M.E., Aras I. (2021). Bond strength of metal brackets to feldspathic ceramic treated with different surface conditioning methods: An in vitro study. Eur. Oral Res..

[27] Mehta A.S., Evans C.A., Viana G., Bedran-Russo A., Galang-Boquiren M.T.S. (2016). Bonding of Metal Orthodontic Attachments to Sandblasted Porcelain and Zirconia Surfaces. Biomed Res. Int..

[28] Xu Z., Li J., Fan X., Huang X. (2018). Bonding Strength of Orthodontic Brackets on Porcelain Surfaces Etched by Er:YAG Laser. Photomed. Laser Surg..

[29] Ahrari F., Heravi F., Hosseini M. (2013). CO_2_ laser conditioning of porcelain surfaces for bonding metal orthodontic brackets. Lasers Med. Sci..

[30] Mirhashemi A., Chiniforush N., Jadidi H., Sharifi N. (2018). Comparative study of the effect of Er:YAG and Er:Cr;YSGG lasers on porcelain: Etching for the bonding of orthodontic brackets. Lasers Med. Sci..

[31] Girish P., Dinesh U., Bhat C.R., Shetty P.C. (2012). Comparison of Shear Bond Strength of Metal Brackets Bonded to Porcelain Surface using Different Surface Conditioning Methods: An in vitro Study. J. Contemp. Dent. Pract..

[32] Lee J.-Y., Kim J.-S., Hwang C.-J. (2015). Comparison of shear bond strength of orthodontic brackets using various zirconia primers. Korean J. Orthod..

[33] Ihsan S.S.A., Mohammed S.A. (2019). Comparison of Shear Bond Strength of Orthodontic Buccal Tube Bonded to Zirconia Crown after Using Two Different (10-MDP)- Containing Adhesive Systems. Int. J. Med. Res. Health Sci..

[34] Costa A.R., Correr A.B., Puppin-Rontani R.M., Vedovello S.A., Valdrighi H.C., Correr-Sobrinho L., Vedovello Filho M. (2012). Effect of bonding material, etching time and silane on the bond strength of metallic orthodontic brackets to ceramic. Braz. Dent. J..

[35] Dalaie K., Mirfasihi A., Eskandarion S., Kabiri S. (2016). Effect of bracket base design on shear bond strength to feldspathic porcelain. Eur. J. Dent..

[36] Kaygisiz E., Egilmez F., Ergun G., Yuksel S., Cekic-Nagas I. (2016). Effect of different surface treatments on bond strength of recycled brackets to feldspathic porcelain. J. Adhes. Sci. Technol..

[37] Topcuoglu T., Oksayan R., Topcuoglu S., Coskun M.E., Isman N.E. (2013). Effect of Er:YAG Laser Pulse Duration on Shear Bond Strength of Metal Brackets Bonded to a Porcelain Surface. Photomed. Laser Surg..

[38] Gonçalves P.R.A., de Moraes R.R., Costa A.R., Correr A.B., Nouer P.R.A., Sinhoreti M.A.C., Correr-Sobrinho L. (2011). Effect of etching time and light source on the bond strength of metallic brackets to ceramic. Braz. Dent. J..

[39] Akpinar Y.Z., Irgin C., Yavuz T., Aslan M.A., Kilic H.S., Usumez A. (2015). Effect of Femtosecond Laser Treatment on the Shear Bond Strength of a Metal Bracket to Prepared Porcelain Surface. Photomed. Laser Surg..

[40] Asiry M.A., Alshahrani I., Hameed M.S., Durgesh B.H. (2018). Effect of Surface Conditioning Protocols and Finishing on Bonding Orthodontic Brackets to Ceramics. J. Biomater. Tissue Eng..

[41] Erdur E.A., Basciftci F.A. (2015). Effect of Ti:sapphire laser on shear bond strength of orthodontic brackets to ceramic surfaces. Lasers Surg. Med..

[42] Asiry M.A., AlShahrani I., Alaqeel S.M., Durgesh B.H., Ramakrishnaiah R. (2018). Effect of two-step and one-step surface conditioning of glass ceramic on adhesion strength of orthodontic bracket and effect of thermo-cycling on adhesion strength. J. Mech. Behav. Biomed. Mater..

[43] Franz A., Raabe M., Lilaj B., Dauti R., Moritz A., Müßig D., Cvikl B. (2019). Effect of two different primers on the shear bond strength of metallic brackets to zirconia ceramic. BMC Oral Health.

[44] Yu J., Zhang Z., Yang H., Wang Y., Muhetaer A., Lei J., Huang C. (2021). Effect of universal adhesive and silane pretreatment on bond durability of metal brackets to dental glass ceramics. Eur. J. Oral Sci..

[45] Abdelnaby Y.L. (2011). Effects of cyclic loading on the bond strength of metal orthodontic brackets bonded to a porcelain surface using different conditioning protocols. Angle Orthod..

[46] Cevik P., Karacam N., Eraslan O., Sari Z. (2017). Effects of different surface treatments on shear bond strength between ceramic systems and metal brackets. J. Adhes. Sci. Technol..

[47] Sarac Y.S., Kulunk T., Elekdag-Turk S., Sarac D., Turk T. (2011). Effects of surface-conditioning methods on shear bond strength of brackets bonded to different all-ceramic materials. Eur. J. Orthod..

[48] Hsu H.-M., Chang C.-J., Liu J.-K. (2015). Effects of Various Acid-Silane Surface Treatments on Bond Strength of Metal Brackets to Porcelain Crowns. J. Med. Biol. Eng..

[49] Durgesh B.H., Alhijji S., Hashem M.I., Kheraif A.A., Malash A.M., Divakar D.D., Shahrani O.A., Asmari M.A., Matinlinna J.P. (2016). Evaluation of an Experimental Silane Primer System in Promoting Adhesion Between Orthodontic Bracket and Ceramic. J. Biomater. Tissue Eng..

[50] Bavbek N.C., Roulet J.-F., Ozcan M. (2014). Evaluation of microshear bond strength of orthodontic resin cement to monolithic zirconium oxide as a function of surface conditioning method. J. Adhes. Dent..

[51] Lima Sandoval P.C., Ratto Tempestini Horliana A.C., Calabró Calheiros F., de Moura-Netto C., Gonçalves F., Antunes Santos A.M., Volpi Mello-Moura A.C. (2020). Evaluation of shear strength in metallic brackets bonded to ceramic surface. Chirurgia (Bucur).

[52] Zarif Najafi H., Oshagh M., Torkan S., Yousefipour B., Salehi R. (2014). Evaluation of the Effect of Four Surface Conditioning Methods on the Shear Bond Strength of Metal Bracket to Porcelain Surface. Photomed. Laser Surg..

[53] García-Sanz V., Paredes-Gallardo V., Bellot-Arcís C., Martínez-León L., Torres-Mendieta R., Montero J., Albaladejo A. (2019). Femtosecond laser settings for optimal bracket bonding to zirconia. Lasers Med. Sci..

[54] Stella J.P.F., Oliveira A.B., Nojima L.I., Marquezan M. (2015). Four chemical methods of porcelain conditioning and their influence over bond strength and surface integrity. Dental Press J. Orthod..

[55] Epperson M., Vazirani N., Armbruster P.C., Kee E.L., Xu X., Ballard R.W. (2021). Hybrid crowns—Bonding protocols and shear bond strength. Australas. Orthod. J..

[56] Ramos T., Lenza M., Reges R., Freitas G. (2012). Influence of ceramic surface treatment on shear bond strength of ceramic brackets. Indian, J. Dent. Res..

[57] Mehmeti B., Haliti F., Azizi B., Kelmendi J., Shahiqi D.I., Jakovljevic S., Milosevic S.A. (2018). Influence of different orthodontic brackets and chemical preparations of ceramic crowns on shear bond strength. Australas. Med. J..

[58] Baeshen H.A. (2021). Influence of photodynamic therapy and different conventional methods on conditioning of lithium di silicate ceramics bonded to metallic brackets: An assessment of bond strength. Photodiagnosis Photodyn. Ther..

[59] Blerim M., Azizi B., Kelmendi J., Iljazi-Shahiqi D., Alar Ž., Anić-Milošević S. (2017). Shear Bond Strength of Orthodontic Brackets Bonded to Zirconium Crowns. Acta Stomatol. Croat..

[60] Yassaei S., Moradi F., Aghili H., Lotfi Kamran M.H. (2013). Shear bond strength of orthodontic brackets bonded to porcelain following etching with Er:YAG laser versus hydrofluoric acid. Orthod. Art Pract. Dentofac. Enhanc..

[61] Hosseini M.H., Sobouti F., Etemadi A., Chiniforush N., Ayoub Bouraima S. (2013). Scanning Electron Microscope Comparative Evaluation of Feldspathic Porcelain Surfaces under Irradiation by Different Powers of Neodymium-Doped Yttrium Aluminium Garnet (Nd:YAG) Laser. J. lasers. Med. Sci..

[62] Alakuş Sabuncuoğlu F., Ertürk E. (2016). Shear bond strength of brackets bonded to porcelain surface: In vitro study. J. Istanbul. Univ. Fac. Dent..

[63] Aksakalli S., Ileri Z., Yavuz T., Malkoc M.A., Ozturk N. (2015). Porcelain laminate veneer conditioning for orthodontic bonding: SEM-EDX analysis. Lasers Med. Sci..

[64] Lestrade A.M., Ballard R.W., Xu X., Yu Q., Kee E.L., Armbruster P.C. (2021). Porcelain surface conditioning protocols and shear bond strength of orthodontic brackets. Australas. Orthod. J..

[65] Poosti M., Jahanbin A., Mahdavi P., Mehrnoush S. (2012). Porcelain conditioning with Nd:YAG and Er:YAG laser for bracket bonding in orthodontics. Lasers Med. Sci..

[66] AlShahrani I., Kamran M.A., Almoammar S., Alhaizaey A. (2019). Photosensitization of lithium di-silicate ceramic by Er, Cr: YSGG and fractional carbon dioxide laser bonded to orthodontic bracket. Photodiagnosis Photodyn. Ther..

[67] May N.B., Araújo É., Vieira L.C.C. (2015). Shear bond strength of ceramic brackets after different pre-treatments in porcelain surface. Brazilian J. Oral Sci..

[68] Alqerban A. (2021). Lithium di silicate ceramic surface treated with Er,Cr:YSGG and other conditioning regimes bonded to orthodontic bracket. Saudi Dent. J..

[69] Elsaka S.E. (2016). Influence of surface treatments on bond strength of metal and ceramic brackets to a novel CAD/CAM hybrid ceramic material. Odontology.

[70] Falkensammer F., Freudenthaler J., Pseiner B., Bantleon H.P. (2012). Influence of surface conditioning on ceramic microstructure and bracket adhesion. Eur. J. Orthod..

[71] Kim J., Park C., Lee J.-S., Ahn J., Lee Y. (2017). The Effect of Various Types of Mechanical and Chemical Preconditioning on the Shear Bond Strength of Orthodontic Brackets on Zirconia Restorations. Scanning.

[72] Alaqeel S.M. (2020). The effect of heat treatment and surface neutralization on bond strength of orthodontic brackets to lithium disilicate glass-ceramic. J. Mech. Behav. Biomed. Mater..

[73] Di Guida L.A., Benetti P., Corazza P.H., Della Bona A. (2019). The critical bond strength of orthodontic brackets bonded to dental glass–ceramics. Clin. Oral Investig..

[74] Martalia C., Anggitia C., Hamid T., Sjamsudin J. (2020). The comparison of shear bond strength of metal orthodontics bracket to porcelain surface using silane and single bond: An in vitro study. J. Int. Oral Health.

[75] Jivanescu A., Bratu D.C., Zaharia R., Marsavina L. (2014). Tensile Bond Strength Evaluation of Two Adhesive Cements Used for Bonding Orthodontic Metal Brackets to Porcelain Fused-to-metal Crowns. Mater. Plast..

[76] Park K.-S., Kim N.-J., Lee C.-J., Park J.-H. (2013). Study on the Shear Bond Strength of Orthodontic Bracket according to the Type of Artificial Tooth Crown and Etching Method. Int. J. Clin. Prev. Dent..

[77] Hellak A., Ebeling J., Schauseil M., Stein S., Roggendorf M., Korbmacher-Steiner H. (2016). Shear Bond Strength of Three Orthodontic Bonding Systems on Enamel and Restorative Materials. Biomed Res. Int..

[78] Grewal Bach G.K., Torrealba Y., Lagravère M.O. (2014). Orthodontic bonding to porcelain: A systematic review. Angle Orthod..

[79] Garboza C.S., Berger S.B., Guiraldo R.D., Fugolin A.P.P., Gonini-Júnior A., Moura S.K., Lopes M.B. (2016). Influence of Surface Treatments and Adhesive Systems on Lithium Disilicate Microshear Bond Strength. Braz. Dent. J..

[80] da Cunha P.F.J.S., Tavares J.G., Spohr A.M., Bellan M.C., Bueno C.H., Cardoso L.I. (2021). Examining the effects of acid etching duration on the bond strength between two CAD/CAM materials and one composite resin. Odontology.

[81] May M.M., Fraga S., May L.G. Effect of milling, fitting adjustments, and hydrofluoric acid etching on the strength and roughness of CAD-CAM glass-ceramics: A systematic review and meta-analysis. J. Prosthet. Dent..

[82] Caparroso Pérez C., Latorre Correa F., Arroyave Hoyos L.J., Grajales Gaviria C.A., Medina Piedrahita V.M. (2014). In vitro evaluation of the effect of hydrofluoric acid concentration and application time on adhesion to lithium disilicate. Rev. Fac. Odontol. Univ. Antioq..

[83] dos Santos D., Bitencourt S., da Silva E., Matos A., Benez G., Rangel E., Pesqueira A., Barão V., Goiato M. (2020). Bond strength of lithium disilicate after cleaning methods of the remaining hydrofluoric acid. J. Clin. Exp. Dent..

[84] Zeidan L.C., Esteves C.M., Oliveira J.A., Brugnera A., Cassoni A., Rodrigues J.A. (2018). Effect of different power settings of Er,Cr:YSGG laser before or after tribosilicatization on the microshear bond strength between zirconia and two types of cements. Lasers Med. Sci..

[85] Feitosa F.A., de Araújo R.M., Tay F.R., Niu L., Pucci C.R. (2017). Effect of high-power-laser with and without graphite coating on bonding of resin cement to lithium disilicate ceramic. Sci. Rep..

